# New Insights Into the Polar Lipid Composition of Extremely Halo(alkali)philic Euryarchaea From Hypersaline Lakes

**DOI:** 10.3389/fmicb.2019.00377

**Published:** 2019-03-12

**Authors:** Nicole J. Bale, Dimitry Y. Sorokin, Ellen C. Hopmans, Michel Koenen, W. Irene C. Rijpstra, Laura Villanueva, Hans Wienk, Jaap S. Sinninghe Damsté

**Affiliations:** ^1^Department of Marine Microbiology and Biogeochemistry, NIOZ Royal Institute for Sea Research, Utrecht University, Texel, Netherlands; ^2^Research Centre of Biotechnology, Winogradsky Institute of Microbiology, Russian Academy of Sciences, Moscow, Russia; ^3^Department of Biotechnology, Faculty of Applied Sciences, Delft University of Technology, Delft, Netherlands; ^4^NMR Spectroscopy, Bijvoet Center for Biomolecular Research, Utrecht University, Utrecht, Netherlands; ^5^Department of Earth Sciences, Faculty of Geosciences, Utrecht University, Utrecht, Netherlands

**Keywords:** halo(alkali)philic, euryarchaea, haloarchaea, glycerol tetraether, membrane spanning lipids, cardiolipin, polar lipid, archaeol

## Abstract

We analyzed the polar membrane lipids of 13 strains of halo(alkali)philic euryarchaea from hypersaline lakes. Nine belong to the class *Halobacteria*, representing two functional groups: aerobic polysaccharide utilizers and sulfur-respiring anaerobes. The other four strains represent halo(alkali)philic methanogens from the class *Methanomicrobia* and a recently discovered class *Methanonatronarchaeia*. A wide range of polar lipids were detected across the 13 strains including dialkyl glycerol diethers (archaeols), membrane-spanning glycerol tetraethers and diether-based cardiolipins. The archaeols contained a range of core lipid structures, including combinations of C_20_ and C_25_ isoprenoidal alkyl chains, unsaturations, and hydroxy moieties. Several diether lipids were novel, including: (a) a phosphatidylglycerolhexose (PG-Gly) headgroup, (b) a *N*,*N*,*N*-trimethyl aminopentanetetrol (APT)-like lipid with a methoxy group in place of a hydroxy group on the pentanetetrol, (c) a series of polar lipids with a headgroup with elemental composition of either C_12_H_25_NO_13_S or C_12_H_25_NO_16_S_2_, and (d) novel cardiolipins containing a putative phosphatidylglycerolphosphate glycerophosphate (PGPGP) polar moiety. We found that the lipid distribution of the 13 strains could be generally separated into two groups, the methanogens (group) and the *Halobacteria* (class) based on the presence of specific core lipids. Within the methanogens, adaption to a high or more moderate salt concentration resulted in different ratios of glycerol dialkyl glycerol tetraethers (GDGTs) to archaeol. The methanogen *Methanosalsum natronophilum* AME2^T^ had the most complex diether lipid composition of any of the 13 strains, including hydroxy archaeol and macrocyclic archaeol which we surmise is an order-specific membrane adaption. The zwitterionic headgroups APT and APT-Me were detected only in the *Methanomicrobiales* member *Methanocalculus alkaliphilus* AMF2^T^ which also contained the highest level of unsaturated lipids. Only alkaliphilic members of the *Natrialbales* order contained PGPGP cardiolipins and the PG-Gly headgroup. The four analyzed neutrophilic members of the *Halobacteria* were characterized by the presence of sulfur-containing headgroups and glycolipids. The presence of cardiolipins with one or more i-C_25_ alkyl chains, generally termed extended archaeol (EXT-AR), in one of the *Methanonatronarchaeia* strains was unexpected as only one other order of methanogenic archaea has been reported to produce EXT-AR. We examined this further by looking into the genomic potential of various archaea to produce EXT-AR.

## Introduction

Extremely halophilic euryarchaea from the class *Halobacteria*, commonly referred to as haloarchaea, have attracted attention both as an object of fundamental research in extremophiles and as producers of extremophilic enzymes with biotechnological potential (Oren, 2002; Begemann et al., 2011; Andrei et al., 2012; Amoozegar et al., 2017). Recently, the knowledge of such euryarchaea has significantly increased by the discovery of anaerobic respiration, the ability to use chitin and cellulose as growth substrates and by the inclusion of relatives outside of the class *Halobacteria* such as methanogens (Sorokin et al., 2015b,c, 2016a,b, 2017a,b, 2018a, 2019). The recent discovery of extremely halo(alkali)philic methanogenic euryarchaea of the class *Methanonatronarchaeia* in hypersaline lakes has provided particular insights into the origin of methanogenesis and the strategies methanogens employ to thrive under hypersaline conditions (Sorokin et al., 2017a). A wide range of haloarchaea has been found to exist in hypersaline NaCl-Na_2_SO_4_-based lakes with neutral pH and alkaline soda lakes with a previously unrecognized potential to utilize native insoluble celluloses and chitin as their sole growth substrate (Sorokin et al., 2015c, 2018a, 2019) and the capability of sulfur respiration in salt-saturated media (Sorokin et al., 2016a,b, 2017b, 2018c).

Sustaining optimal membrane function is crucial for all microorganisms in order to maintain cell homeostasis, but is a particular challenge in extreme environments such as hypersaline lakes. The characteristic membrane lipids of haloarchaea form the barrier between the cell's internal functions and the harsh outside environment. In general, the membrane lipids of Archaea are characterized by the presence of ether linkages between a glycerol moiety and isoprene-based alkyl chains. Archaeal membrane lipids include *sn*-2,3-diphytanyl glycerol diether with two C_20_ isoprenoid chains (archaeol, AR), extended archaeol (EXT-AR, with a C_20_ and C_25_ isoprenoid chain) and tetraethers with two glycerol moieties connected by two C_40_ isoprenoid chains [glycerol dialkyl glycerol tetraethers (GDGT)]. Membrane-spanning GDGTs can contain up to eight cyclopentane moieties (i.e., GDGT-n, where *n* is the number of cyclopentane moieties). Membrane-spanning GDGTs have not been detected in members of the class *Halobacteria*. Archaea also produce bisphosphatidylglycerol diethers, analogous to the diacyl cardiolipins found in Bacteria and Eukarya (Corcelli et al., 2000; Lattanzio et al., 2002; Corcelli, 2009; Angelini et al., 2012; Yoshinaga et al., 2012). The membrane polar lipids of halophilic archaea, mostly from the class *Halobacteria*, have been characterized in a wide range of studies (Kushwaha et al., 1975; Kates et al., 1993; de Souza et al., 2009; Angelini et al., 2012; Dawson et al., 2012; Lobasso et al., 2015). Phosphatidylglycerophosphate methyl ester (PGP-Me) has often found to be the dominant polar headgroup. It is thought that the bulky, double charged nature of this headgroup contributes to membrane stability at high salt concentrations (Tenchov et al., 2006). Indeed, Kellermann et al. (2016) observed that for the neutrophilic haloarchaeon *Haloferax* (*H.) volcanii*, there was a correlation between the amount of PGP-Me and the concentration of Mg^2+^ in the media during culturing experiments. Kellermann et al. (2016) hypothesized that the terminal methyl group of PGP-Me acts as an antenna, while the closely positioned, negatively charged neighboring phosphate moieties allow PGP-Me to bind divalent cations [as opposed to monovalent cation bonding to phosphatidylglycerol (PG) or cardiolipins, cf. Figure 9 of Kellermann et al. (2016)]. They suggested that by up- or downregulating the number of negative charges at its surface, a cell can balance membrane permeability under varying salt concentrations. Indeed, the most important membrane adaption for haloarchaea is low permeability to water, protons and monovalent cations (i.e., Na^+^ and K^+^) (Oger and Cario, 2013). Other phospho-based polar headgroups have been detected in haloarchaea in lesser proportions along with some glyco- and sulfo-lipids (Kates, 1992; Koga and Morii, 2005). Diether lipids are advantageous in extreme environments such as hypersaline conditions, and are considered to be a critical part of the “salt-in” strategy generally employed by haloarchaea to cope with extreme salinity, by which they accumulate a high intracellular concentration of KCl, while excluding Na^+^ (Oren, 1999). In particular, the presence of unsaturated AR and EXT-AR are characteristic of haloarchaea (Dawson et al., 2012; Oger and Cario, 2013). Indeed, Kellermann et al. (2016) observed for *H. volcanii* an increase in the degree of unsaturation with increasing Na^+^ levels particularly within AR with a PG headgroup (PG-AR). However, Kellermann et al. (2016) noted that the total pool of AR in *H. volcanii* did not exhibit the same trend in unsaturation, which they suggested was due to impaired growth performance at both the low (1 M Na^+^) and high (5 M Na^+^) ends of the salt concentration range. While lipid analysis is an essential tool for understanding archaeal membrane composition, culturing conditions are known to affect this composition (Elling et al., 2015; Kellermann et al., 2016) and thus it is also useful to examine lipid synthesis genes (e.g., Villanueva et al., 2014) to determine not only the present but also the dormant lipid species within an organism's inventory.

**Figure 1:**
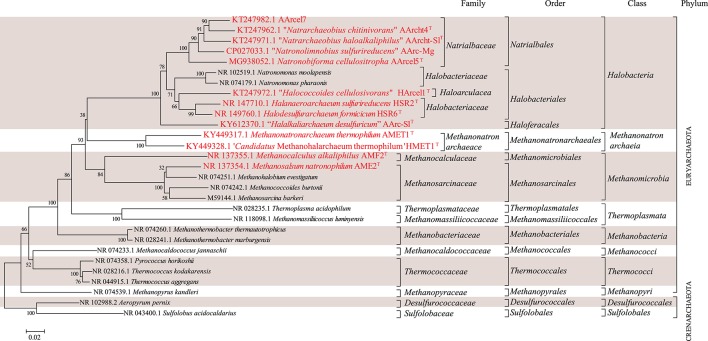
16S-rRNA-based phylogenetic tree showing position of the investigated halo(alkali)philic strains amongst other archaeal lineages. Bootstrap test values (1,000 replicates) are shown next to the branches. Bar scale indicates number of base substitutions per site. Colored boxes are a visual aid.

In this study the polar lipid composition of 13 strains of halo(alkali)philic euryarchaea (Table 1; Figure 1) isolated from different hypersaline lakes was analyzed using High Performance Liquid Chromatography-Mass Spectrometry (HPLC-MS) and Nuclear Magnetic Resonance (NMR) spectroscopy. Among them are four methanogenic species, of which two (*Methanonatronarchaeum* (*Mn*.) *thermophilum* AMET1^T^ and “*Candidatus* (*Ca*.) Methanohalarchaeum (Mh.) thermophilum” HMET1) are extremely halophilic methyl-reducers forming a novel class *Methanonatronarchaeia* (Sorokin et al., 2017a, 2018b), while the other two (*Methanosalsum* (*Ms*.) *natronophilum* AME2^T^ and *Methanocalculus* (*Mc*.) *alkaliphilus* AMF2^T^) are haloalkaliphiles from soda lakes (Sorokin et al., 2015b). Haloalkaliphiles from soda lakes are not strictly halophilic and their name is applied to discriminate them from freshwater alkaliphiles. *Mc. alkaliphilus* AMF2^T^ can be regarded as a moderately salt-tolerant alkaliphile (growth up to 2 M total Na^+^), while *Ms. natronophilum* AME2^T^ is an extremely salt-tolerant alkaliphile (growth up to 4 M total Na^+^). Five haloarchaeal isolates are aerobic polysaccharide utilizers: cellulolytic “*Halococcoides* (*Hc*.) *cellulosivorans”* HArcel1^T^, *Natronobiforma* (*Nb*.) *cellulositropha* AArcel5 and uncharacterised strain AArcel7 and two natronarchaeal chitinolytic strains “*Natrarchaeobius* (*Na*.) *chitinivorans”* AArcht4^T^ and “*Na. haloalkaliphilus”* AArcht-S1^T^ are members of the orders *Halobacteriales* and *Natrialbales* in the class *Halobacteria*. The last four strains belong to a group of anaerobic sulfur-respiring haloarchaea. Two halophilic strains comprise two novel genera in the family *Halobacteriaceae*: *Halanaeroarchaeum* (*Hn*.) *sulfurireducens* HSR2^T^, which grows exclusively by acetate-dependent sulfur respiration (Sorokin et al., 2016a,b), and *Halodesulfurarchaeum* (*Hd*.) *formicicum* HSR6^T^, which is capable of utilizing either formate or hydrogen as their electron donor, and elemental sulfur, thiosulfate or dimethylsulfoxide as electron acceptors (Sorokin et al., 2017b). The two other strains, AArc-Mg and AArc-S1, are alkaliphilic haloarchaea, capable of growth by dissimilatory sulfur reduction [provisionally belonging to a new species “*Natronolimnobius* (*Nl*.) *sulfurireducens*” (*Natrialbales*) and a new genus and species “*Halalkaliarchaeum* (*Ha*.) *desulfuricum*” (*Haloferacales*) (Sorokin et al., 2018c)]. While the dominant lipid groups have been previously reported for a number of these strains, our work reports the full range of dialkyl glycerol diether and tetraether membrane lipids present in the 13 strains, including the in-depth structural characterization of novel compounds.

**Table 1:** Strains of halophilic euryarchaea examined in this study.

	**Strain**	**Species**	**Growth conditions**					
			**pH**	**Na^**+**^ M**	**K^**+**^ mM**	**Mg^**2+**^ mM**	**T ^**°**^C**	**O_**2**_ conditions**
Methanogens	AMET1^T^	*Methanonatronarchaeum* (*Mn*.) *thermophilum*	9.5	4.0	5.0	1.0	48	Anaerobic
	HMET1	“*Ca*. Methanohalarchaeum (Mh.) thermophilum”	7.0	4.0	5.0	1.0	50	Anaerobic
	AME2^T^	*Methanosalsum* (*Ms*.) *natronophilum*	10	3.0	na	1.0	30	Anaerobic
	AMF2^T^	*Methanocalculus* (*Mc*.) *alkaliphilus*	10	0.6	na	1.0	30	Anaerobic
Anaerobic sulfur reducers	HSR2^T^	*Halanaeroarchaeum* (*Hn*.) *sulfurireducens*	7.0	4.0	5.0	1.0	37	Anaerobic
	HSR6^T^	*Halodesulfurarchaeum* (*Hs*.) *formicicum*	7.0	4.0	5.0	1.0	37	Anaerobic
	AArc-Mg	“*Natronolimnobius* (*Nl*.) *sulfurireducens*”	10	4.0	5.0	1.0	37	Anaerobic
	AArc-Sl^T^	“*Halalkaliarchaeum* (*Ha*.) *desulfuricum*”	9.1	4.0	5.0	1.0	37	Anaerobic
Polysaccharide utilizers Cellulolytics	HArcel1^T^	“*Halococcoides* (*Hc*.) *cellulosivorans*”	7.0	4.0	5.0	1.0	37	Aerobic
	AArcel5^T^	*Natronobiforma* (*Nb*.) *cellulositropha*	9.5	4.0	5.0	1.0	37	Aerobic
	AArcel7	unpublished	9.0	4.0	5.0	1.0	37	Aerobic
Chitinolytics	AArcht4^T^	“*Natrarchaeobius* (*Na*.) *chitinivorans*”	9.5	4.0	5.0	1.0	37	Aerobic
	AArcht-Sl^T^	“*Natrarchaeobius* (*Na*.) *haloalkaliphilus*”	9.3	4.0	5.0	1.0	37	Aerobic

^*^description submitted to IJSEM. Na, not applicable.

*For class, order and family of strains see Figure 1*.

## Materials and Methods

### Culturing

*Mc. alkaliphilus* AMF2^T^ was grown at 0.6 M total Na^+^ and 30°C with formate as electron donor and acetate as carbon source and *Ms. natronophilum* AME2^T^ at 2 M total Na^+^ with methanol as a sole substrate, both at pH 9.5, as described previously (Sorokin et al., 2015a,b). The other 11 extremely halophilic archaeal isolates were grown at 4 M total Na^+^, 5 mM KCl and 1 mM Mg^2+^ until late exponential growth phase. *Mn. thermophilum* AMET1^T^ and “*Ca*. Mh. thermophilum” HMET1 were grown anaerobically with methanol and formate (50 mM each) and yeast extract as carbon source (100 mg L^−1^) at 50°C and pH either 9.5 (2 M Na^+^ as carbonates and 2 M NaCl) or 7.0 (4 M NaCl), respectively (Sorokin et al., 2018b). Sulfur-reducing neutrophilic haloarchaea were grown anaerobically with sulfur as the electron acceptor and either 10 mM pyruvate (*Hn. sulfurireducens* HSR2^T^) or 50 mM formate (*Hd. formicicum* HSR6^T^) as the electron donor and 200 mg L^−1^ of yeast extract as carbon source at 37°C as described by Sorokin et al. (2016a,b, 2017b). Alkaliphilic sulfur reducing haloarchaea were cultivated anaerobically with sulfur as electron acceptor at 37°C with 10 mM butyrate as the electron donor at pH 9.8 (strain AArc-Mg) or with 20 mM pyruvate at pH 9.2 (strain AArc-Sl^T^) (Sorokin et al., 2018c). Aerobic alkaliphilic polysaccharide utilizers were grown either with 10 mM cellobiose at pH 9.5 (strains AArcel5^T^ and AArcel7) or 10 mM N-acetylglucosamine (strains AArcht4^T^ and AArcht-Sl^T^) at pH 9.5 and 9.1, respectively, as described previously (Sorokin et al., 2015c, 2018a, 2019). All cultures were grown in duplicate and the final biomass was pooled together in a single sample for lipid analysis.

### Hydrolysis of Biomass and Core Lipid Analysis

In order to remove the headgroups from the intact polar lipids (IPLs) and to obtain the remaining core lipids the freeze-dried biomass was hydrolyzed with HCl/MeOH (1.5 N) by refluxing for 3 h. The hydrolysate was adjusted with aqueous KOH to pH 5, extracted three times with dichloromethane, and dried over Na_2_SO_4_.

#### HPLC-MS Analysis of GDGTs and AR

The hydrolyzed biomass extracts were redissolved in hexane:isopropanol (99:1, v:v) and filtered through a 0.45 μm PTFE filter and analyzed using HPLC-atmospheric pressure chemical ionization/mass spectrometry (HPLC-APCI/MS) on an Agilent 1100/Hewlett Packard 1100 MSD instrument equipped with automatic injector and HP-Chemstation software according to Hopmans et al. (2000) with the following modifications. Separation was achieved in normal phase with two Prevail Cyano columns in series (150 × 2.1 mm; 3 μm) with a starting eluent of hexane:propanol (99.5:0.5, v:v) and a flow rate of 0.2 mL min^−1^. This remained isocratic for 5 min followed by a linear gradient to 1.8% propanol at 45 min. The injection volume was 10 μL. An external standard of AR:GDGT-0 (1:1, w:w) run in sequence before and after the samples was used to quantify AR and GDGT-0. GDGT-1 was assumed to have the same response as GDGT-0 and was in this way semi-quantified. Hereafter, the concentrations of AR and GDGTs are expressed as percent of their total combined concentration.

#### GC-MS Analysis of Mono and Diethers

Hydrolyzed biomass extracts were methylated with diazomethane in diethyl ether which was removed under a stream of N_2_ and trimethylsilylated using N,O-bis(trimethylsilyl)trifluoroacetamide (BSTFA) and pyridine (1:1; 20 min at 60°C). Gas chromatography (GC) was performed using a Hewlett Packard 6890 gas chromatograph equipped with an on-column injector and a flame ionization detector (FID). A fused silica capillary column (CP Sil5 25 × 0.32 mm, *df* = 0.12 μm) with helium as a carrier gas was used. The samples were injected at 70°C. The GC oven temperature was subsequently raised to 130°C at a rate of 20°C min^−1^, and to 320°C at 4°C min^−1^. The temperature was then held constant for 15 min. Additionally, all hydrolyzed fractions were analyzed by GC–MS for compound identification. The structural designation of lipids was evaluated by the comparison of their retention times and mass spectral fragmentation patterns with standards and with published spectra (e.g. Pancost et al., 2001). GC–MS was conducted using Thermo Finnigan Trace Ultra GC connected to ThermoFinnigan DSQ MS operated at 70 eV, with a mass range m/z 50–800 and 3 scans s^−1^ with a silica column (CP Sil-5, 25 × 0.32 mm) and He as the carrier gas at a constant flow rate of 2 mL min^−1^. The oven temperature program initiated at 70°C, increased first at a rate of 20°C min^−1^ to 130°C, and next at a rate of 4°C min^−1^ to the final temperature of 320°C, which was held for 10 min.

GC peak area response (FID) of the mono and diethers was recorded. One of the diethers, AR, had previously been quantified (by HPLC-MS, section HPLC-MS analysis of GDGTs and AR). The two separate hydrolysis-derived ether lipid data sets (GC-MS derived and HPLC-MS derived), were both normalized to AR and combined to give an estimate of the total ether distribution (in percent of the total, Table 2). Combining the data sets involved several assumptions, such as that AR had been conserved equally during the two analyses and that the response of the ethers during GC-MS analysis was uniform. Hence this combined data set should be examined with these caveats in mind.

**Table 2:** The distribution of ether lipids released by acid hydrolysis (presented as % of total) across the 13 strains of haloarchaea with three different types of physiology.

**Core lipid**		**Methanogens**				**Anaerobic sulfur reducers**				**Polysaccharide utilizers**				
										**Cellulolytics**		**Chitinolytics**	
		**AMET1^**T**^**	**HMET1**	**AME2^**T**^**	**AMF2^**T**^**	**HSR2^**T**^**	**HSR6^**T**^**	**AArc-Mg**	**AArc-Sl^**T**^**	**HArcel1^**T**^**	**AArcel5^**T**^**	**AArcel7**	**AArcht4^**T**^**	**AArcht-Sl^**T**^**
Phytanyl glycerol monoethers^a^	*sn*-2-C_20_	1		4	2		0.2		4	3	0.4	0.2	0.5	
	*sn*-3-C_20_	1		12	10		0.5		0.4	0.3	0.5	0.6	0.4	
Extended (sesterterpenyl) glycerol monoethers^a^	*sn*-2-C_25_						0.5				0.8	2	0.6	
	*sn*-3-C_25_									2				49
Archaeol^b^	C_20−20_	32	20	45	73	47	40	44	88	81	58	12	56	51
Extended archaeols^a^	*sn*-2-C_25_		1			53	59	56			40	85	43	
	*sn*-3-C_25_								7	13				
Macrocyclic archaeol ^a^				6										
OH-Archaeol^a^				15										
GDGT-0^b^		59	79	17	15									
GDGT-1^b^		7		1	0.1									

*Full strain names given in Table 1. GDGT, glycerol dialkyl glycerol tetraether (where n is the number of cyclopentane moieties), OH, hydroxy. This distribution is produced by combining two separate data sets (^a^GC derived and ^b^HPLC-MS derived), both normalized to archaeol. Note that, due to assumptions made when combining the two data sets (see Section GC-MS analysis of mono and diethers) this should be treated as an estimation of the total ether lipid distribution. sn-n-C_XX_, n denotes the sn position of the alkyl chain and xx refers to the number of isoprenoidal carbons. For sn-n-C_25_ of the two extended archaeols, n denotes the sn position of the extended alkyl chain (sesterterpenyl)*.

### IPL Extraction and Analysis

Lipids were extracted from freeze-dried biomass using a modified Bligh-Dyer procedure (Schouten et al., 2008). Briefly, the biomass was treated ultrasonically three times for 10 min with a solvent mixture of methanol, dichloromethane and phosphate buffer (2:1:0.8, v:v:v). After sonication, the combined supernatants were phase-separated by adding additional dichloromethane and buffer to a final solvent ratio of 1:1:0.9 (v:v:v). The organic phase containing the IPLs was collected and the aqueous phase re-extracted two times with dichloromethane. Finally, the combined extract was dried under a stream of N_2_ gas. Before analysis, the extract was redissolved in a mixture of n-hexane:2-propanol:water (72:27:1, v:v:v) at a concentration of 2 mg mL^−1^ and aliquots were filtered through 0.45 μm regenerated cellulose syringe filters (4 mm diameter; Grace Alltech, Deerfield, IL).

#### HPLC-ITMS

For general IPL screening, the IPL extracts were analyzed by high performance liquid chromatography-ion trap mass spectrometry (HPLC-ITMS) according to Sturt et al. (2004) with modifications as described previously (Sinninghe Damsté et al., 2011). The analysis was performed on an Agilent 1,200 series HPLC (Agilent, San Jose, CA), equipped with thermostatted auto-injector and column oven, coupled to an LTQ XL linear ion trap with Ion Max source with electrospray ionization (ESI) probe (Thermo Scientific, Waltham, MA). Separation was achieved on a LiChrospher diol column (250 × 2.1 mm, 5 μm particles; Alltech) maintained at 30°C. The following elution program was used with a flow rate of 0.2 mL min^−1^: 100% A for 1 min, followed by a linear gradient to 66% A: 34% B in 17 min, maintained for 12 min, followed by a linear gradient to 35% A: 65% B in 15 min, where A = hexane/2-propanol/formic acid/14.8 M NH_3aq_ (79:20:0.12:0.04, v:v:v:v) and B = 2-propanol/water/formic acid/ 14.8 M NH_3aq_ (88:10:0.12:0.04, v:v:v:v). The lipid extract was analyzed by an MS routine where a positive ion scan (*m/z* 400–2,000) was followed by a data dependent MS^2^ experiment where the base peak of the mass spectrum was fragmented (normalized collision energy (NCE) 25, isolation width 5.0, activation Q 0.175). This was followed by a data-dependent MS^3^ experiment where the base peak of the MS^2^ spectrum was fragmented under identical fragmentation conditions. This process was repeated on the second to fourth most abundant ions of the initial mass spectrum. Identification of the IPLs was carried out by comparison of their masses and their diagnostic fragmentations in MS^2^ with those described in the literature (e.g., Ferrante et al., 1987; Kates et al., 1993; Yoshinaga et al., 2011).

Intact polar lipids (IPLs) were quantified in terms of their MS peak area response. As different IPLs show different response behavior, the relative abundance of peak area does not necessarily reflect the actual relative abundance of the different IPLs. However, this method allows for comparison between the strains analyzed in this study, especially as they all contained at least one, but generally three, lipid groups in common. The peak areas were determined from extracted ion chromatograms of the combined [M+H]^+^, [M+NH_4_]^+^, and [M+Na]^+^ ion (where present) for each individual IPL species. The peak areas for the individual species were summed for data handling in groups based on headgroups and/or core lipids.

#### UHPLC-HRMS

Additional analysis of selected extracts was carried out using an ultra-high performance liquid chromatography-high resolution mass spectrometry (UHPLC-HRMS) method (Besseling et al., 2018) in order to determine the accurate mass of certain unknown compounds. An Agilent 1290 Infinity I UHPLC equipped with thermostatted auto-injector and column oven, coupled to a Q Exactive Orbitrap MS with Ion Max source with heated electrospray ionization (HESI) probe (Thermo Fisher Scientific, Waltham, MA) was used. Separation was achieved on an YMC-Triart Diol-HILIC column (250 × 2.0 mm, 1.9 μm particles, pore size 12 nm; YMC Co., Ltd, Kyoto, Japan) maintained at 30°C. The eluent composition and flow rate were the same as described above for the Agilent 1200/LTQ XL ion trap HPLC-MS system although the elution program was adjusted thus: 100% A, followed by a linear increase to 30% B at 20 min, followed by a 15 min hold, and a further increase to 60% B at 50 min. Positive ion ESI settings were: capillary temperature, 275°C; sheath gas (N_2_) pressure, 35 arbitrary units (AU); auxiliary gas (N_2_) pressure, 10 AU; spray voltage, 4.0 kV; probe heater temperature, 275°C; S-lens 70 V. Target lipids were analyzed with a mass range of *m/z* 350–2,000 (resolving power 70,000 at *m/z* 200), followed by data-dependent tandem MS^2^ (resolving power 17,500), in which the 10 most abundant masses in the mass spectrum were fragmented successively (stepped normalized collision energy 15, 22.5, 30; isolation width 1.0 *m/z*). The Q Exactive was calibrated within a mass accuracy range of 1 ppm using the Thermo Scientific Pierce LTQ Velos ESI Positive Ion Calibration Solution. During analysis dynamic exclusion was used to temporarily exclude masses (for 6 s) in order to allow selection of less abundant ions for MS^2^.

### Compound Isolation and NMR Analysis

Specific IPLs were isolated from Bligh-Dyer extracts of *Mc. alkaliphilus* AMF2^T^ biomass using an Agilent Technologies (Santa Clara, CA) 1100 series HPLC with a thermostatted autoinjector, column oven, and a Foxy Jr. fraction collector (Teledyne Isco, Lincoln, NE, USA). Aliquots of filtered Bligh-Dyer extract (200 μL) were injected onto a LiChrospher DIOL column (250 mm x 10 mm, 10 μm: Alltech, Deerfield, IL), all fractions were eluted using the identical gradient and mobile phase composition described above for the HPLC-ITMS analysis, but at a flow rate of 3 mL min^−1^. The column effluent was collected in 3 mL fractions. Fractions from semi-preparative HPLC were screened for the presence of the target compound with flow injection analysis using HPLC-ITMS as described above, and the fractions containing the target compound were pooled. For further purification the combined fractions were re-injected and column effluent was collected in 15 s fractions.

*N*,*N*,*N*,-trimethyl aminopentanetetrol (APT)-AR and an unknown lipid (referred to below as **IIa)** were dissolved in 500 μL 99.9% CDCl_3_ at concentrations of 3.0 and 0.8 mg ml^−1^, and NMR experiments were performed at 298 K on a Bruker 900-MHz Avance III spectrometer equipped with 5 mm TCI cryoprobe, running under TOPSPIN 3.2. One-dimensional (1D) ^1^H and ^1^H-decoupled 1D ^13^C and Distortionless Enhancement by Polarization Transfer (DEPT)-135 experiments were recorded with spectral widths/offsets of 20 ppm/4.7 ppm, 200 ppm/100 ppm, and 200 ppm/100 ppm and with 16,384 (16 k), 4,096 (4 k), and 4,096 (4 k) complex points, respectively. 12- by 12-ppm 2D correlation spectroscopy (COSY), total correlation spectroscopy (TOCSY), and nuclear Overhauser effect spectroscopy (NOESY) experiments were performed with 512 by 256 complex points (by 200 for the NOESY) and an offset frequency of 4.7 ppm. Mixing times were 60 and 120 ms for TOCSY and 250 ms for NOESY experiments. (^1^H, ^13^C)-heteronuclear single-quantum correlation spectroscopy (HSQC) and (^1^H, ^13^C)-heteronuclear multiple-bond correlation spectroscopy (HMBC) experiments were recorded with spectral widths/offsets of 12/4.7 ppm for protons, 200/100 ppm for ^13^C HSQC (512 x 64 complex points) and 150/75 ppm for the HMBC (1 k × 80 complex points). All spectra were calibrated with respect to internal residually protonated CHCl_3_ at 7.24 ppm (^1^H) and 77.0 ppm (^13^C).

### Genomic and Phylogenetic Analyses

Partial 16S rRNA gene sequences of the strains mentioned in the manuscript and others were obtained from the Silva 128 release (https://www.arb-silva.de/). A phylogenetic tree of 16S rRNA gene sequences was inferred with the Neighbor-Joining method (Saitou and Nei, 1987) in MEGA6 (Tamura et al., 2013). The evolutionary distances were computed using the Jukes-Cantor method (Jukes and Cantor, 1969). The analysis involved 29 nucleotide sequences with a total of 1,185 positions in the final dataset.

Putative polyprenyl synthase coding genes were identified in the available genomes of the studied strains along with a selection of other archaea with PSI-BLAST (Position-Specific iterated BLAST) searches at the protein level (www.ncbi.com) using two iteration steps using the annotated geranylfarnesyl disphosphate synthase of *Aeropyrum* (*A*.) *pernix* [accession number Q9UWR6.1; (Tachibana et al., 2000)] as query sequence and limiting the search to hits with percentage of identity higher than 20% and *e*-value <1 × 10^−30^. Putative polyprenyl synthases were aligned by Muscle (Edgar, 2004) in the MEGA6 software (Tamura et al., 2013) and edited manually. Phylogenetic reconstruction was performed by maximum likelihood in the MEGA 6 software (Tamura et al., 2013) based on the JTT matrix-based model (Jones et al., 1992). A discrete Gamma distribution was used to model evolutionary rate differences among sites (5 categories, +G parameter = 1.5942). The rate variation model allowed for some sites to be evolutionarily invariable ([+*I*], 4.2308% sites). The tree was drawn to scale, with branch lengths measured in the number of substitutions per site. The analysis involved 49 amino acid sequences. There was a total of 421 positions in the final dataset.

## Results

Thirteen strains of haloarchaea isolated from hypersaline soda lakes were compared for the composition of their membrane lipids. The strains were separated into three groups based on their physiology: 4 methanogens, 5 polysaccharide utilizers and 4 sulfur-respiring haloarchaea (Table 1).

### Core Lipid Composition

The ether lipids detected by GC-MS and HPLC-APCI/MS were all normalized to AR and combined to give the estimated distribution of all hydrolysis-derived ether core lipids (Table 2). In 12 of the 13 strains, AR was either a dominant (73–88%) or major (20–58%) component, whereas in one polysaccharide utilizing, moderately alkaliphilic strain, AArcel7, AR was only present as a minor component (12%). Two forms of EXT-AR in both C_20−25_ and C_25−20_ configurations (with different *sn*2 and *sn*3 arrangements) were detected. *Ms. natronophilum* AME2^T^ was found to contain hydroxy archaeols (OH-AR; 15%) and the macrocyclic AR (MAR; 6%), neither of which were detected in any other strains. Four different phytanyl glycerol monoethers were detected, the *sn*2- and *sn*3- alkyl glycerol monoether, in both the C_20_ and C_25_ forms. These did not constitute more than 16% of the diethers, except in the case of moderately alkaliphilic chitinolytic strain “*Na*. *haloalkaliphilus”* AArcht-Sl^T^, where C_25_ (extended) *sn*-3-alkyl glycerol ether was present as 49% of the diether fraction. We have interpreted the four alkyl glycerol monoethers as artifacts that are formed during acid hydrolysis from diether lipids containing unsaturated alkyl moieties (de Souza et al., 2009) as confirmed by the IPL measurements (see section IPLs). GDGT-0 and GDGT-1 were detected in the methanogenic strains, but not in the members of *Halobacteria*. GDGT-0 accounted for 59 and 79% of the total core lipids in the two extremely halophilic methanogens from the class *Methanonatronarchaeia*, alkaliphilic *Mn. thermophilum* AMET1^T^ and neutrophilic “*Ca*. Mh. thermophilum” HMET1, while GDGT-1 was 7% in the former but absent in the latter. In the two haloalkaliphilic members of the class *Methanomicrobia, Ms. natronophilum* AME2^T^ and *Mc. alkaliphilus* AMF2^T^, GDGT-0 accounted for 17 and 15% while GDGT-1 was present at 1 and <0.1%, respectively.

### IPLs

**Figure 2:**
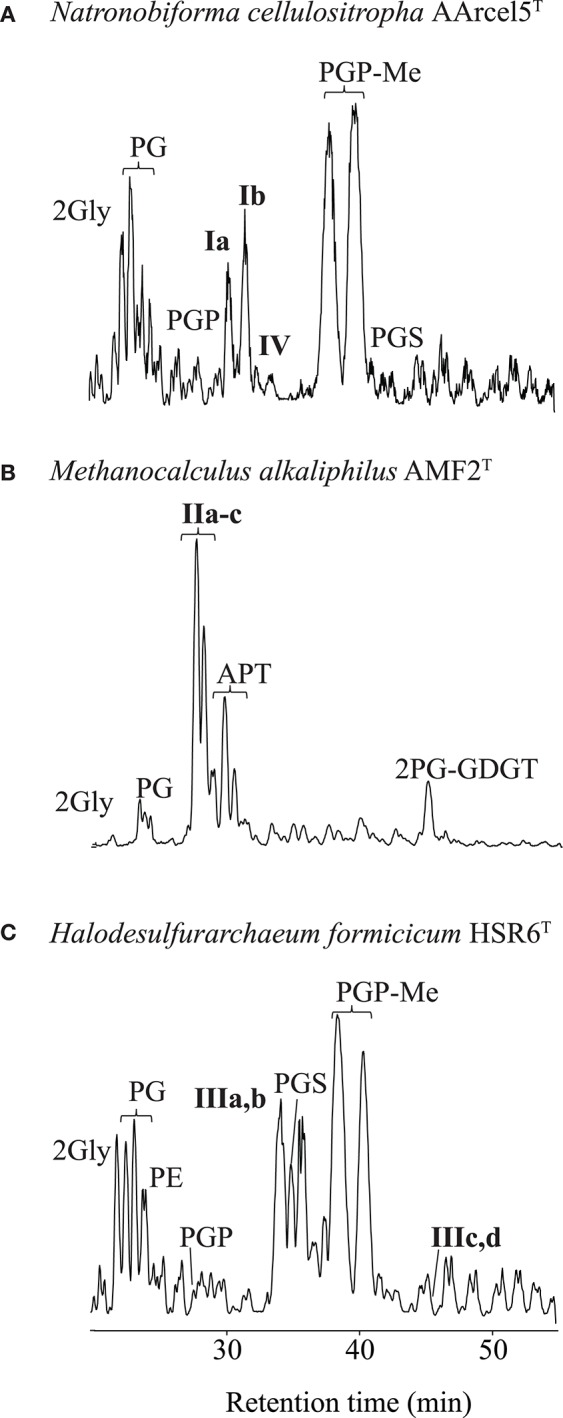
Partial base peak chromatogram (Gaussian smoothed) of the HPLC-ITMS analysis of IPLs in three strains of haloarchaea: **(A)** Natronobiforma cellulositropha AArcel5, **(B)** Methanocalculus alkaliphilius AMF2T, **(C)** Halodesulfurarchaeum formicicum HSR6T. For abbreviations of IPL names see text.

Analysis by HPLC-ITMS (e.g., Figure 2) and UHPLC-HRMS of the Bligh-Dyer extracts of the 13 strains resulted in the identification of a wide range of IPLs, including novel compounds. We describe these in three sections: diethers, ether analogs of cardiolipins and GDGTs.

#### Diether IPLs

**Figure 3:**
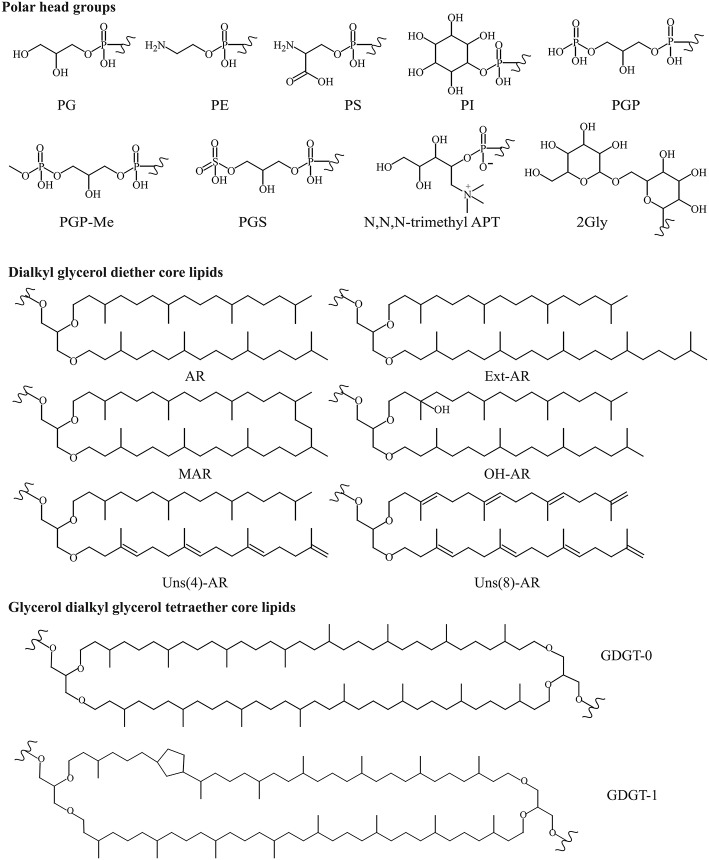
Structures of known polar headgroups and core lipids described in this study. Polar headgroups: PE, phosphatidylethanolamine; PS, phosphatidylserine; PI, phosphatidylinositol; PG, phosphatidylglycerol; PGP, phosphatidylglycerophosphate; PGP-Me, phosphatidylglycerophosphate methyl ester; PGS, phosphatidylglycerosulfate; trimethyl APT, *N*,*N*,*N*-trimethyl aminopentanetetrol (for other methylations see Figure 5); 2Gly, dihexose. Dialkyl glycerol diether core lipids; AR, archaeol; EXT-AR, extended archaeol; MAR, macrocyclic archaeol; OH-AR, hydroxy archaeol; Uns, unsaturated. Glycerol dialkyl glycerol tetraether core lipids (GDGTs, where *n* is the number of cyclopentane moieties).

Diether IPLs detected by HPLC-ITMS (see Table S1 for all species detected, structures in Figure 3) contained the following polar headgroups: phosphatidylethanolamine (PE), phosphatidylglycerol (PG), phosphatidylglycerophosphate (PGP), and its methyl ester (PGP-Me), APT [with tri-, di- and mono-N-methylations), phosphatidylinositol (PI), phosphatidylserine (PS), phosphatidylglycerosulfate (PGS), and dihexose (2Gly, also known as DGA-1 in e.g., Kates (1992)]. The core component of the IPLs included AR, EXT-AR, OH-AR, and MAR (also detected by GC-MS analysis), unsaturated (Uns) forms of both AR and EXT-AR (with between 1 and 8 unsaturations) and OH-AR (with between 1 and 5 unsaturations). The diether IPLs (Table S1) were examined in terms of their MS peak area response, which is generally dependent on polar group ionization and therefore do not reflect the absolute quantities of the different lipids. Besides these known diether species, also a number of novel diether lipids (**I–III**, Figure 2) were detected. Their identification using combinations of MS^2^ fragmentation, accurate mass and NMR spectroscopy is described below.

##### Novel diether lipids Ia and Ib

**Figure 4:**
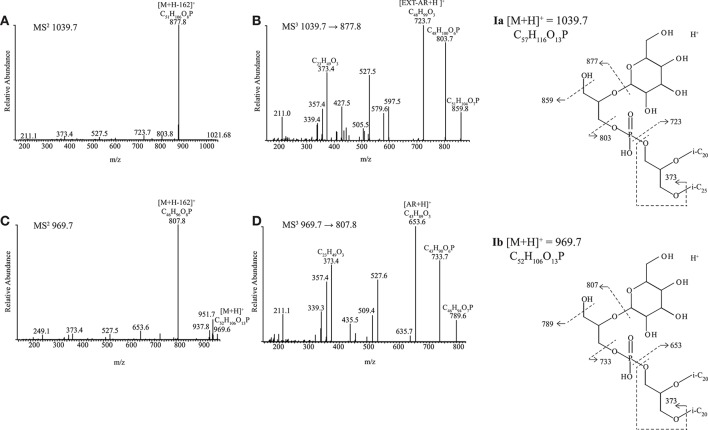
HPLC-ITMS^2^ and MS^3^ spectra with inserts of structure with fragmentations indicated for **(A,B) Ia** PG-Gly-EXT-AR and **(C,D) Ib** PG-Gly-AR. i-C_20_, C_20_ isoprenoid chain; i-C_25_, C_25_ isoprenoid chain.

Two of the alkaliphilic polysaccharide utilizing strains, *Nb. cellulositropha* AArcel5^T^ and strain AArcel7 contained lipids (e.g., **Ia and Ib**, Figure 2) with parent ions appearing in MS spectra at *m/z* 1039.7 and 969.7, respectively. For both parent ions, upon MS^2^ fragmentation, a loss of 162 Da (loss of hexose) was observed, generating product ions at *m/z* 877.8 (Figure 4A) and 807.8 (Figure 4C), respectively. These product ions match the *m/z* value of the protonated molecules ([M+H]^+^; Table S1) of PG-EXT-AR and PG-AR, respectively. Indeed, upon further fragmentation in MS^3^, product ions representing the EXT-AR (*m/z* 723.7, Figure 4B) and AR (*m/z* 653.6, Figure 4D) core lipids were observed. Therefore, we identified these lipids as phosphatidylglycerohexose-EXT-AR (PG-Gly-EXT-AR, **Ia**) and phosphatidylglycerohexose-AR (PG-Gly-AR, **Ib**). This headgroup combined with a diacyl core lipid has been described in halophilic gammaproteobacterium of the genus *Halomonas* (Giordano et al., 2007), while a very similar diether phosphoglycolipid attached to a sulfated sugar moiety was identified in the alkaliphilic haloarchaeon *Natrononomonas* (*N*.) *moolapensis* (Hoffmann et al., 2015). The position of the sugar of both the bacterial and archaeal glycosylated-PG described in the literature were determined by NMR to be at the 2-glycero position (Giordano et al., 2007; Hoffmann et al., 2015). In our study the MS^2^ fragmentation pattern could not confirm whether the hexose was at the 1- or 2- glycerol position, but the similarity of the PG-Gly spectra with those of 1-(6-sulfo-D-glcp/galf-β1,2-glycero)-phospho-2,3-diphytanylglycerol found in *N. moolapensis* (Hoffmann et al., 2015) provides an indication that they both have same structural arrangement.

##### Novel N,N,N-trimethyl APT derivatives

**Figure 5:**
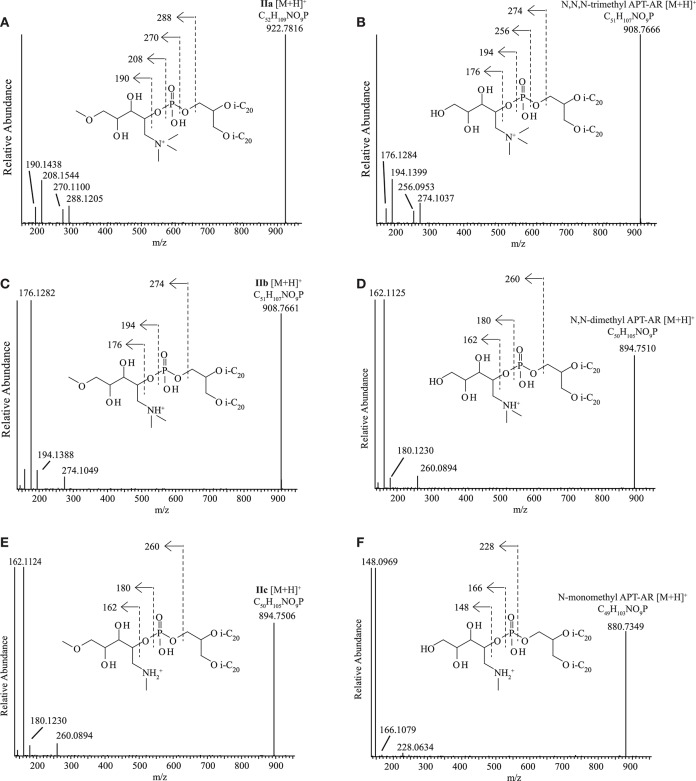
UHPLC-HRMS MS^2^ spectra with structures and fragmentations indicated for **(A)** *N*,*N*,*N*-trimethyl aminomethoxypentanetriol (trimethyl APT-Me, **IIa**), **(B)** *N*,*N*,*N*-trimethyl aminopentanetetrol (trimethyl APT), **(C)** *N*,*N*-dimethyl aminomethoxypentanetriol (dimethyl APT-Me, **IIb**), **(D)** *N*,*N*-dimethyl aminopentanetetrol (dimethyl APT), **(E)** N-monomethyl aminomethoxypentanetriol (monomethyl APT-Me, **IIc**), **(F)** N-monomethyl aminopentanetetrol (monomethyl APT). i-C_20_, C_20_ isoprenoid chain.

The dominant but unknown IPL in *Mc. alkaliphilus* AMF2^T^ (**IIa**, Figure 2) exhibited a parent ion at *m/z* 923.0 during HPLC-ITMS analysis. Upon UHPLC-HRMS analysis it was found to have an accurate mass of 922.7816 (Table S2). Upon UHPLC-HRMS fragmentation, product ions at *m/z* 288.1205, 270.1100, 208.1544, and 190.1438 were formed (Figure 5A; Table S2). Both the parent ion and all the main fragments produced are offset by 14 Da compared to the parent ion and fragments of *N*,*N*,*N*-trimethyl APT-AR ([M+H]^+^ *m/z* 908.7666, Table S2). These fragment ions of *N*,*N*,*N*-trimethyl APT-AR (Figure 5B) can be interpreted as follows: *m/z* 274.1037 is the intact *N*,*N*,*N*-trimethyl APT headgroup, *m/z* 256.0953 is a loss of H_2_O from the *m/z* 274.1037 ion, *m/z* 194.1399 is the headgroup without the phosphate moiety, and *m/z* 176.1284 represents a loss of H_2_O from the *m/z* 194.1399 ion. The mass difference between fragments of *N*,*N*,*N*-trimethyl APT and of **IIa** was always in the range 14.0145–14.0168 Da which indicates that **IIa** contains an additional CH_2_ group (14.0151 Da) in its headgroup, likely by conversion of one of the alcohol groups into a methoxy group, i.e., *N*,*N*,*N*-trimethyl aminopentanemethoxytriol (APT-Me). Based on this identification, we interpreted the MS^2^ spectrum of **IIa** (Figure 5A; Table S2) as follows: *m/z* 288.1205 is the methylated *N*,*N*,*N*-trimethyl APT headgroup with the phosphate moiety, *m/z* 270.1100 is loss of H_2_O from the *m/z* 288.1205 ion, *m/z* 208.1544 is the *N*,*N*,*N*-trimethyl APT-Me headgroup without the phosphate moiety, and *m/z* 190.1438 is loss of H_2_O from the *m/z* 208.1544 ion. A series of mono- to octa- Uns-AR with a *N*,*N*,*N*-trimethyl APT-Me headgroup (HPLC-ITMS [M+H]^+^ from *m/z* 920.9–906.7) exhibited very similar HPLC-ITMS MS^2^ fragmentations (Table S1) with a decreasing intensity of the product ion arising from loss of one alkyl chain, depending on the number of unsaturations. The minor unknowns **IIb, IIc** were identified using UHPLC-HRMS as *N*,*N*-dimethyl (*m/z* 908.7661, Figure 5C) and N-monomethyl APT-Me-AR (*m/z* 894.7506, Figure 5E), analogous to *N*,*N*-dimethyl APT-AR (*m/z* 894.7510, Figure 5D) and N-monomethyl APT-AR (*m/z* 880.7349, Figure 5F).

In order to confirm the identification of the novel *N*,*N*,*N*-trimethyl APT-Me derivatives **IIa-c** and to assess the position of the Me group on the pentanetetrol moiety, the most abundant IPL of this series, **IIa**, was purified using semi-preparatory HPLC in order to carry out analysis by NMR spectroscopy. The putative *N*,*N*,*N*-trimethyl APT-AR was also purified and analyzed by NMR spectroscopy for comparison. Both *N*,*N*,*N*-trimethyl APT-AR and IPL **IIa** exhibited similar NMR spectra with almost identical ^13^C- and ^1^H-NMR shifts for most of carbon and proton atoms, including those of the polar headgroup (Table S3). The ^13^C- and ^1^H-shifts associated with the glycerol unit and the phytanyl chains were identified in comparison with those described for GDGTs (Sinninghe Damsté et al., 2002) and are not presented here. The ^13^C- and ^1^H-NMR shifts reported by Ferrante et al. (1987) for *N*,*N*,*N*-trimethyl APT are similar to ours and the few differences that occur can all be accounted for by the differences in solvent and experimental conditions used (e.g., ^2^H_2_O and benzene-*d*_6_/methanol-*d*_4_ vs. CDCl_3_ used in this study). Whereas, the *N*,*N*,*N*-trimethyl APT headgroup exhibits 6 carbon signals for its polar headgroup, that of the putative *N*,*N*,*N*-trimethyl APT-Me IPL exhibited seven carbon signals, confirming that the additional 14 Da was due to an additional C atom and its associated protons. This additional C atom (C_7_; Table S3) was determined by a DEPT experiment to be either a primary or tertiary carbon. HSQC analysis provided evidence for a single signal at 3.36 ppm, indicative of a primary methyl group. Furthermore, its ^13^C shift at 59 ppm agrees with a methoxy group and hence it was assigned to be an *N*,*N*,*N*-trimethyl APT-Me. The carbon and associated proton signals of three carbon atoms (C_1_, C_2_, C_6_) of the *N*,*N*,*N*-trimethyl APT-Me headgroup were almost identical to those of *N*,*N*,*N*-trimethyl APT-AR (Table S3), which provides evidence the additional CH_3_ group (C_7_) was bound to an oxygen atom at either C_3_, C_4_, or C_5_. The HMBC spectrum provided confirmation that C_7_ was in fact bound to the oxygen at C_5_ as the protons at C_7_ interacted most strongly with those of C_5_ (see structure insert Table S3). Further confirmation of the structure from the HMBC spectrum were the cross peaks between the protons of C_6_ with those of C_1_, and those of C_1_ with both C_6_ and C_3_ and from the COSY spectrum that indicated that the protons of C_2_ were *J*-coupled with C_3_.

##### Diethers with novel sulfur-containing headgroups (IIIa-d)

**Figure 6:**
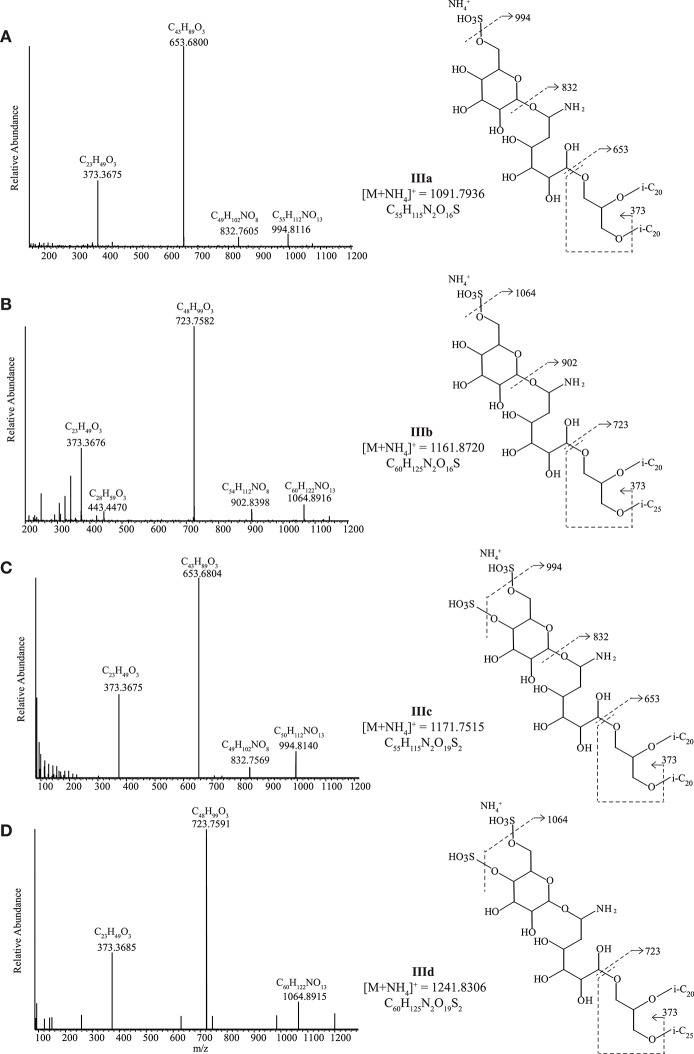
UHPLC-HRMS MS^2^ spectra with putative structures and fragmentations of the C_6_H_23_NO_13_S headgroup with **(A)** an AR core (**IIIa**) and **(B)** an EXT-AR core (**IIIb**) and the C_6_H_23_NO_16_S_2_ headgroup with **(C)** an AR core (**IIIc**) and **(D)** an EXT-AR core (**IIId**). i-C_20_, C_20_ isoprenoid chain; i-C_25_, C_25_ isoprenoid chain.

Four unknown IPLs with two closely related headgroups were detected in four strains (all neutrophilic) distributed across all three archaeal metabolic groups, namely in methanogenic ‘*Ca*. Mh. thermophilum' HMET1, cellulolytic “*Hc. cellulosivorans”* HArcel1^T^ and sulfur-respiring *Hd. formicicum* HSR6^T^ and *Hn. sulfurireducens* HSR2^T^. During HPLC-ITMS analysis, two compounds with the same headgroup, **IIIa**, and **IIIb** produced [M+NH_4_]^+^ ions at *m/z* 1091.5 and 1161.5, and two with a closely related headgroup (**IIIc** and **IIId**) produced [M+NH_4_]^+^ at *m/z* 1171.4 and 1241.4 (Figure 6). Structural identification using UHPLC-HRMS resulted in the partial elucidation of the structure of these unknown headgroups (accurate masses of the parent ions and fragments are given in Table S4). The MS^2^ spectrum (Figure 6A) of **IIIa** possessing a [M+NH_4_]^+^ of *m/z* 1091.7936 ion exhibited a loss of SO_3_ and NH_3_ from the [M+NH_4_]^+^ to produce a fragment ion at *m/z* 994.8116. The ion at *m/z* 832.7605 was interpreted as the loss of hexose (C_6_H_10_O_5_, 162.0523 Da) from the ion at *m/z* 994.8116. A further loss of C_6_H_10_NO_5_ from the *m/z* 832.7605 ion produced the most abundant product ion at *m/z* 653.6800 representing the AR core lipid). The fragment ion *m/z* 373.3675 was assigned as a phytanyl moiety attached to a glycerol moiety (C_23_H_49_O_3_). The elemental composition of this compound, taken in combination with its MS^2^ fragmentation allowed us to propose a putative structure of **IIIa** as having a headgroup with elemental composition C_12_H_25_NO_13_S and an AR core (Figure 6A; Table S4). The second IPL with the same headgroup, **IIIb**, possessed a [M+NH_4_]^+^ ion at *m/z* 1161.8720 and exhibited similar losses in its MS^2^ spectrum (Figure 6B and Table S4): loss of SO_3_ and NH_3_ from the [M+NH_4_]^+^ to produce a fragment ion at *m/z* 1064.8916, sequential losses of hexose and a C_6_H_10_NO_5_ group to produce ions at *m/z* 902.8398 and 723.7582, respectively. The smaller fragment ions were assigned a phytanyl moiety attached to a glycerol moiety (*m/z* 373.3676, C_23_H_49_O_3_) and the corresponding C_25_ isoprenoid alkyl chain attached to a glycerol moiety (*m/z* 443.4470, C_28_H_59_O_3_). Based on accurate mass determination of fragments and losses the **IIIb** compound was identified as having a C_12_H_25_NO_13_S headgroup with an EXT-AR core.

**IIIc** and **IIId** produced similar MS^2^ spectra to **IIIa** and **IIIb** (Figures 6C,D and Table S4), but **IIIc** and **IIId** contained an additional SO_3_H group (based on UHPLC-HRMS^2^ fragmentation, Table S4) on the polar headgroup. Hence, they were assigned as a C_12_H_25_NO_16_S_2_ headgroup with either an AR (Figure 6C) or EXT-AR (Figure 6D) core. The exact position(s) of the SO_3_H group(s) could not be established. Biomass was not available in great enough quantity to carry out the compound isolation that would be required for NMR analysis.

#### Ether Analogs of Cardiolipins

**Figure 7:**
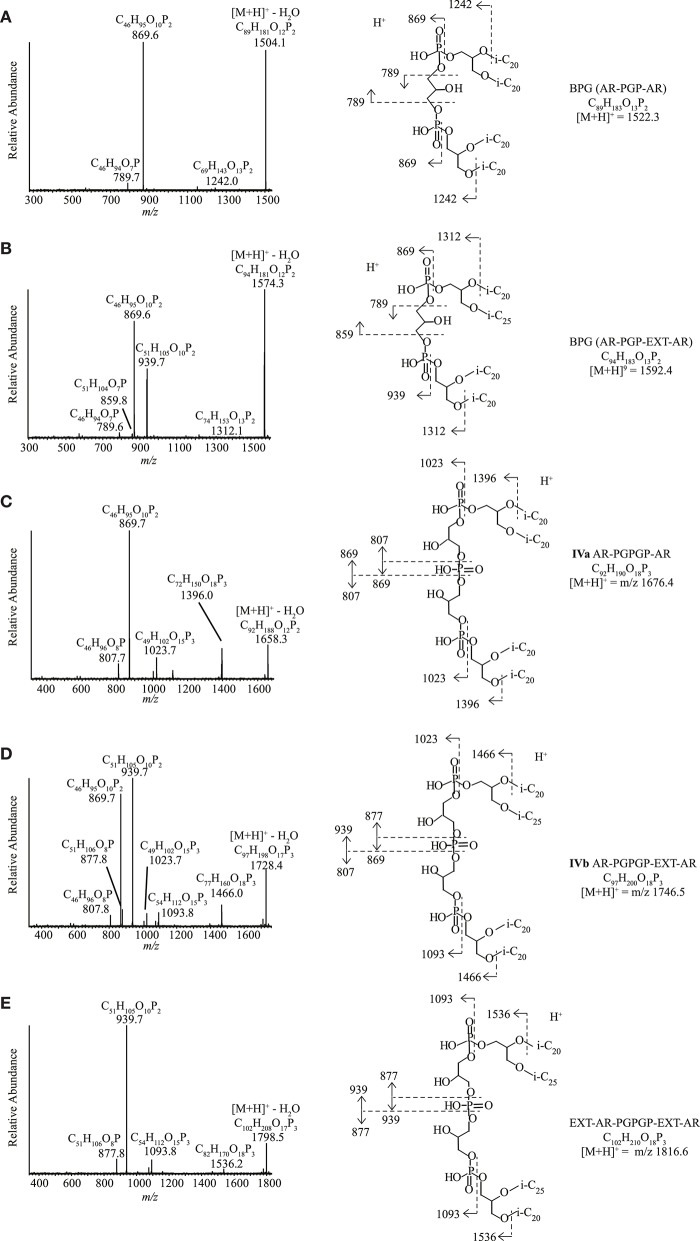
HPLC-ITMS^2^ spectra of cardiolipins with putative structures and fragmentations. **(A)** PGP-AR, AR, **(B)** PGP-AR, EXT-AR, **(C)** PGPGP-AR, AR, **(D)** PGPGP-AR, EXT-AR, and **(E)** PGPGP-EXT-AR, EXT-AR. i-C_20_, C_20_ isoprenoid chain; i-C_25_, C_25_ isoprenoid chain.

Ether analogs of cardiolipins, with two dialkyl glycerol diether cores connected by a polar moiety, were detected in four of the 13 strains (Tables 3, S1). Two of these cardiolipins were identified as bisphosphatidylglycerols (BPGs) containing two AR cores (Figure 7A) and a BPG with an AR and an EXT-AR core (Figure 7B). These IPLs have been reported previously in other haloarchaea (Lattanzio et al., 2002; Corcelli, 2009; Angelini et al., 2012; Lobasso et al., 2015). Three novel tetraether cardiolipins (**IVa-c**) were found in three polysaccharide utilizing alkaliphilic haloarchaeal strains, “*Na. chitinivorans*” AArcht4^T^, *Nb. cellulositropha* AArcel5^T^ and AArcel7. They exhibited [M+H]^+^ masses (*m/z* 1676.4, 1746.5, 1816.6) identical to those of a series of unknown cardiolipins from the cell membrane of two species of alkaliphilic haloarchaea from the alkaliphilic genus *Natronococcus* that were previously reported by Angelini et al. (2012). While Angelini et al. (2012) postulated these were glycocardiolipins, their MS^2^ spectra did not support the presence of sugar groups. One of our three novel tetraether cardiolipins (**IVa**) exhibited a [M+H]^+^ at *m/z* 1676.4. Its MS^2^ spectrum (Figure 7C) contained product ions at *m/z* 1396.0, consistent with a loss of a phytanyl chain (280 Da) and at *m/z* 1023.7 (loss of AR, 653 Da). The most abundant ion at *m/z* 869.7 is also present in the MS^2^ spectrum of AR-PGP-AR (Figure 7A). In the case of AR-PGP-AR the *m/z* 869.7 represents a loss of AR (653 Da; Figure 7A), whereas for **IVa** it represents a loss of 807 Da. This additional 154 Da within the polar bridge of the cardiolipin can be explained by an additional glycerophosphate moiety. Hence, we have assigned **IVa** as containing a putative phosphatidylglycerophosphate glycerophosphate (PGPGP) polar group (Figure 7C). Based on previous nomenclature (e.g., Lattanzio et al., 2002; Corcelli, 2009; Angelini et al., 2012), the novel cardiolipins would be termed bisphosphatidylglycerol glycerophosphates (BPGGPs). A similar tetraether cardiolipin (**IVb**) exhibited a [M+H]^+^ at *m/z* at 1746.5, 70 Da larger than **IVa**. This difference in mass suggests the presence of an EXT-AR core instead of an AR core. Indeed, the product ions in the MS^2^ spectrum arising from fragmentation of this ion mirrored the losses seen for **IVa** cardiolipin with addition of losses associated with an EXT-AR chain (Figure 7D). These were: *m/z* 1466.0 (loss of a phytyl chain, 280 Da), *m/z* 1023.7, and 1093.8 (loss of AR, 653 Da and EXT-AR, 723 Da, respectively) and four ions at *m/z* 807.8, 869.7, 877.8, and 939.7, which represent the AR and EXT-AR containing halves of the parent ion, fragmented on either side of the central phosphate group (Figure 7D). Furthermore, the most dominant product ions, at *m/z* 869.6 and 939.7 were also the dominant ions in the MS^2^ spectrum of PGP-AR, EXT-AR (Figure 7B). The [M+H]^+^ of the third novel cardiolipin (**IVc**) was 140 Da larger than the first, at *m/z* 1816.6, in agreement with the presence of two EXT-ARs instead of a single AR-group. The product ions in its MS^2^ spectrum are interpreted as follows: *m/z* 1536.2: loss of a phytanyl chain of 280 Da, *m/z* 1093.8: loss of EXT-AR of 723 Da, two ions at *m/z* 877.8 and 939.7, representing the two halves of the parent ion when fragmented at either side of the central phosphate group (Figure 7E). As for compounds **IIIa-d**, NMR analysis was not carried out as there was not sufficient biomass for compound isolation.

#### GDGT-Based IPLs

We detected membrane-spanning GDGTs in 4 out of the 13 examined halo(alkali)philic euryarchaea, all methanogens (Table 3). These included 2Gly-GDGT-0, PG-GDGT-0-PG, PG-GDGT-1-PG, and PG-GDGT-0-PE, identified by comparison of their masses and their diagnostic fragmentation patterns in MS^2^ with those described in the literature (Rossel Cartes, 2009; Yoshinaga et al., 2011).

**Table 3:** Distribution of different classes of intact polar lipids (% of total peak area response) for the 13 strains of haloarchaea.

**IPL sType**	**Headgroup**	**Methanogens**				**Anaerobic sulfur reducers**				**Polysaccharide utilizers**				
										**Cellulolytics**			**Chitinolytics**	
		**AMET1^**T**^**	**HMET1**	**AME2^**T**^**	**AMF2^**T**^**	**HSR2^**T**^**	**HSR6^**T**^**	**AArc-Mg**	**AArc-Sl^**T**^**	**HArcel1^**T**^**	**AArcel5^**T**^**	**AArcel7**	**AArcht4^**T**^**	**AArcht-Sl^**T**^**
Diether	IIIa-d		9			17	14			16				
	2Gly		29		2	6	0.1			1	3			
	PGP	0.2	5	1		1	0.2	1	1	1	1	2	0.4	3
	PG	28	40	23	4	20	11	33	31	28	28	42	32	30
	PE	1		1		2	3						1	
	PS	8		5										
	APT-Me (IIa-c)				58									
	APT				23									
	PGS					8	22			4				
	PGP-Me	30	3	30		46	49	67	68	51	46	47	57	68
	PI			38										
	PG-Gly (Ia,b)										21	4		
Cardiolipin	PGP (BPG)		6											
	PGPGP (IVa-c)										1	5	9	
GDGT	2Gly		8											
	2PG	30		1	13									
	PG/PE	2												

*Full strain names given in Table 1. IPLs were examined in terms of their peak area response from HPLC-ITMS analysis and thus the proportions reported do not necessarily reflect the quantitative relative abundance. PE, phosphatidylethanolamine; PS, phosphatidylserine; PI, phosphatidylinositol; PG, phosphatidylglycerol; PGP, phosphatidylglycerophosphate; PGP-Me, phosphatidylglycerophosphate methyl ester; PGS, phosphatidylglycerosulfate; APT, aminopentanetetrol; APT-Me, aminomethoxypentanetriol; PG-Gly, phosphatidylglycerohexose; 2Gly, dihexose; PGPGP, phosphatidylglycerophosphate glycerophosphate; BPG, bisphosphatidylglycerol; GDGT, glycerol dialkyl glycerol tetraether (where n is the number of cyclopentane moieties). For data handling the three methylation of APT-Me-AR and the three methylations of APT-AR were combined as one group. For both APT-Me-AR and APT-AR the N,N,N-trimethyl form was the most abundant (minimum of total peak area 94% for APT-Me-AR and 96% for APT-AR), the N,N-dimethyl APT-Me-AR and APT-AR were present in a low relative abundance (maximum of total peak area 6% for APT-Me-AR and 2.5% for APT-AR) while the N-monomethyl APT-Me-AR and APT-AR were the lowest in relative abundance (maximum of total peak area < 0.5% for APT-Me-AR and 1.5% for APT-AR)*.

## Discussion

A wide range of intact polar dialkyl glycerol diethers, membrane-spanning GDGTs and cardiolipins were identified in 13 strains of halo(alkali)philic archaea in this study (cf. Table S1). Many of the intact polar diethers detected have been described previously in other haloarchaea. However, some IPLs described in this study have not, to the best of our knowledge, been previously reported. These include an APT-Me polar headgroup, a series of cardiolipins with a PGPGP polar group, a PG-Gly headgroup with an AR core, and AR and EXT-AR combined with headgroups containing a hexose, a C_6_H_10_NO_5_ sugar with either one (**IIIa,b**) or two (**IIIc,d**) sulfate groups.

### Factors Determining Lipid Composition

We examined the distribution of membrane lipids across the 13 strains and examined what factors determined their lipid composition. Within the scope of this project it was not possible to carry out controlled culture experiments with varying growth conditions. Hence, here we are limited to hypothesizing on the factors controlling the lipid distributions at fixed growth conditions optimal for each examined strain. Previous studies have examined extensively the impact of phylogeny and physiology on the membrane lipid composition of archaea (Koga et al., 1998; Koga and Nakano, 2008; Elling et al., 2015; Kellermann et al., 2016). It is known that low permeability to water, protons and monovalent cations (i.e., Na^+^ and K^+^) is the most important membrane adaption for haloarchaea (Oger and Cario, 2013). The adaption of haloarchaeal membranes to salt, temperature, oxygen levels, and UV light examined experimentally by Kellermann et al. (2016) allowed them to hypothesize about the function of e.g., different polar headgroups (PG, PGP-Me, and cardiolipins) and core lipid composition. For example, they proposed that by changing the proportion of these polar headgroups, and hence the number of negative charges at its membrane surface, a haloneutrophile can actively balance membrane permeability under varying salt concentrations.

**Figure 8:**
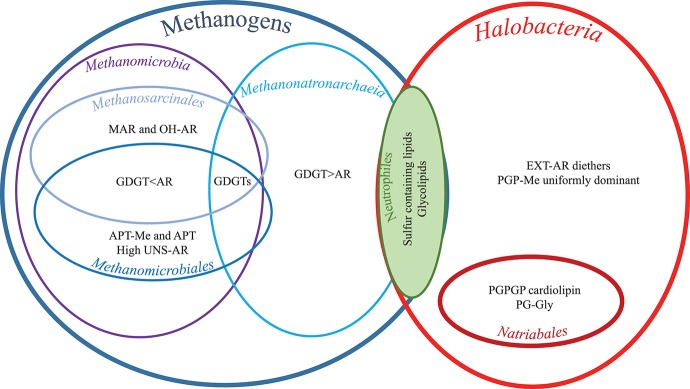
Characteristic lipids as grouped by physiology, phylogenetic orders and classes. For full species names and for groups, orders, and classes see Table 1. For lipid abbreviations see text.

Hereafter, we examine specific lipids and groups of lipids and discuss the possible factors they are associated with, including the phylogenetic relationships between the strains (cf. Figure 1 and Table 1), the three different metabolic processes the 13 strains carry out (methanogenesis, polysaccharide utilization and sulfur respiration) and possible lipid adaptions associated with growth conditions. We have summarized the characteristic lipids associated with these separate factors in Figure 8 and we discuss them in detail in the following sections.

#### Characteristic Lipids of the Methanogens

The only characteristic shared by all four methanogens, and not by the other nine strains (all *Halobacteria*), was the presence of membrane-spanning GDGT lipids (Table 2). This is to be expected since GDGTs have been identified in a wide range of archaea including methanogenic euryarchaea, but not so far in the *Halobacteria* class [cf. summary tables in Koga et al. (1998) and Schouten et al. (2013)]. PG-GDGT-0-PG and PG-GDGT-1-PG were found in three of the four methanogenic strains (Tables 2, S1). 2Gly-GDGT-0 was only detected in neutrophilic “*Ca*. Mh. Thermophilum” HMET1 (Table S1). 2Gly-GDGTs, including 2Gly-GDGT-0, have been found previously in a wide range of environments (but not in soda lakes), including peat bogs, a suboxic water column and various marine benthic ecosystems (Lipp et al., 2008; Rossel et al., 2008; Lipp and Hinrichs, 2009; Schubotz et al., 2009; Liu et al., 2010; Yoshinaga et al., 2012; Gibson et al., 2013), and while 2Gly-GDGT-0 has been identified in cultures of *Methanobacterium thermoautotrophicum* (Koga et al., 1993), it has also been found in *Thaumarchaeota* (Schouten et al., 2008; Pitcher et al., 2011). As we discuss further in section Neutrophilic strains, the presence of this sugar headgroup may relate to the neutrophilic nature of HMET1 (cultured at pH 7, Table 1). PG-GDGT-0-PE was only found in *Mn. thermophilum* ${\text{AMET1}}_{,}^{\text{T}}$ grown optimally at moderately thermophilic conditions in addition to salinity and pH extremes. PG-GDGT-PE has been previously described in two methane-rich seep environments, where it was specifically associated with anaerobic methane-oxidizing archaea of the ANME-1 cluster (Rossel et al., 2011).

Within the methanogens there was a division between the two classes based on the ratio of GDGTs to AR (cf. Figure 8). The two members of *Methanonatronarchaeia* (“*Ca*. Mh. thermophilum” HMET1 and *Mn. thermophilum* AMET1^T^) contained more GDGT than AR (GDGT0+GDGT1/AR = 4 and 2, respectively) while the two members of the class *Methanomicrobia* (*Ms. natronophilum* AME2^T^ and *Mc. alkaliphilus* AMF2^T^) contained more AR than GDGTs (ratio values 0.4 and 0.2, respectively). This difference may be an adaption to external salt concentration: while the two *Methanonatronarchaeia* are extremely halophilic methanogens (both cultured at 4 M Na^+^), the *Methanomicrobia* are haloalkaliphiles, in that *Ms. natronophilum* AME2^T^ is an extremely salt-tolerant alkaliphile (cultured at 2 M total Na^+^), while *Mc. alkaliphilus* AMF2^T^ is a moderately salt-tolerant alkaliphile (cultured at 0.6 M total Na^+^). The presence of GDGTs in the membrane of methanogens may be associated with the reduction of wasteful ion cycling under chronic energy stress conditions (Valentine, 2007), as tetraether-containing membranes are less permeable to ions than purely diether-based membranes (Mathai et al., 2001; Shinoda et al., 2005).

The polar headgroup PS was present in *alkaliphilic members of Methanonatronarchaeia* (*Mn. thermophilum* AMET1^T^) and *Methanosarcinales* (*Ms. natronophilum* AME2^T^), but it was absent in any members of the *Halobacteria*. This polar headgroup has been described previously in a wide range of methanogenic archaea (Koga et al., 1993, 1998; Koga and Morii, 2005), and its presence in *Ms. natronophilum* AME2^T^, a member of *Methanosarcinales*, and absence in *Mc. alkaliphilus* AMF2^T^, belonging to *Methanomicrobiales*, agrees with the findings of Koga et al. (1998). While PI was only detected in *Methanosarcinales* member *Ms. natronophilum* AME2^T^, this polar headgroup has been identified across a wide range of phyla and is not specific to methanogenic euryarchaea (Koga et al., 1993; Koga and Morii, 2005; Oger and Cario, 2013). However, the combination of the PI headgroup with the OH-AR and Uns(1-5)-OH-AR cores (cf. Table S1) is much less common. IPLs containing OH-AR and Uns(n)-OH-AR have been detected in deep-sea cold seep sediments (Yoshinaga et al., 2011), in the crenarchaeon *Sulfolobus* (*S*.) *acidocaldarius* (Sprott et al., 1997), the methanogen *Methanosarcina* (*M*.) *barkeri* (Nishihara and Koga, 1991) and in the extreme cold-adapted methanogen *Methanococcoides* (*M*.) *burtonii* (Nichols et al., 2004). *M. barkeri, M. burtonii*, and *Ms. natronophilum* AME2^T^ are all members of the *Methanosarcinales* order, which suggests that OH-AR and Uns(n)-OH-AR may be characteristic of this order rather than being related to e.g., cold adaption.

Two cardiolipins based on a PGP polar group and generally termed BPGs (Corcelli, 2009; Angelini et al., 2012) were detected only in “*Ca*. Mh. thermophilum” HMET1 from the class *Methanonatronarchaeia* (Table 3). To the best of our knowledge BPGs have only been described to date in haloarchaea from the order *Halobacteriales* (Lattanzio et al., 2002; Corcelli, 2009; Angelini et al., 2012; Lobasso et al., 2015; Kellermann et al., 2016; although not in the *Halobacteriales* investigated in this study), but not before in any methanogenic members of the Euryarchaeota. Our results indicate that synthesis of BPGs is not restricted to the *Halobacteriales*. Indeed, Yoshinaga et al. (2012) detected novel lyso-cardiolipins (with only two isoprenoid chains) in surface sediments of methane-charged deep-ocean seeps which are usually dominated by uncultured anaerobic methanotrophic (ANMEs) archaea related to methanogenic euryarchaea, in particularly from the order *Methanosarcinales* (Knittel and Boetius, 2009). It is interesting to note that unlike the other 3 methanogens, and the majority of the other strains (cf. section Neutrophilic strains), “*Ca*. Mh. thermophilum” HMET1 is a haloneutrophile, being cultured at pH 7. Previously, the haloneutrophile *H. volcanii* has been shown experimentally to adapt to low Mg^2+^ levels (as per the culturing conditions here, e.g., 1 mM Mg^2+^) by upregulating cardiolipin levels, in order to counterbalance the absence of “cardiolipin-surrogates” that form at higher Mg^2+^ concentrations, between the Mg^2+^ cations and two anionic PG lipids (Kellermann et al., 2016).

#### Characteristic Lipids of the Halobacteria

The absence of membrane-spanning GDGT lipids in *Halobacteria* has been hypothesized to have evolved to effectively respire using mechanisms acquired from bacteria through horizontal gene transfer (Kellermann et al., 2016). The authors suggest that the specific structure of haloarchaeal bilayer membranes has evolved to provide optimal balance between preventing ion permeability and maintaining sufficient fluidity for respiratory electron transport.

While diether lipids of the four methanogenic strains contained no EXT-AR (with the exception of an EXT-AR cardiolipin in '*Ca*. Mh. thermophilum' HMET1, see section Biosynthesis of EXT-AR), many of the analyzed *Halobacteria* strains showed a high proportion of EXT-AR in agreement with the other cultured genera from the class (Tindall et al., 1984; Teixidor et al., 1993; Tachibana, 1994; Xu et al., 1999, 2001; Gibson et al., 2005; Xue et al., 2005; Wang et al., 2007; Minegishi et al., 2010). This absence of diether EXT-AR in methanogens is in agreement with previous studies (e.g., Koga et al., 1998). HPLC-ITMS analysis (Table S1) revealed that the *Halobacteria* core lipids also contained Uns-EXT-AR. The presence of EXT-AR has been suggested to provide a “zipper” like effect between the two layers of the membrane, interlinking the C_25_ isoprenoid of the “outward” facing lipid, with those of the “inward” facing lipids (De Rosa et al., 1982) providing increased membrane stability. Kellermann et al. (2016) hypothesized that in the case of alkaliphilic haloarchaea (six of the nine *Halobacteria* strains examined here) the EXT-AR zipper configuration within the bilayer is not compatible with membrane-shielding PG-cation interactions (as seen in neutrophilic haloarchaea). Therefore, alkaliphilic haloarchaea with high levels of EXT-AR would need to upregulate the amount of PGP-Me, to provide an alternative mode of Mg^2+^ buffering at the membrane surface. Indeed, in agreement with this idea, PGP-Me was the dominant headgroup (46–68%, Table 3) across the *Halobacteria* and has been described previously as a distinguishing feature of haloarchaea (Kushwaha et al., 1981; Kates, 1992; Kates et al., 1993; Sprott et al., 1997; Lattanzio et al., 2002; Oren, 2002; Gibson et al., 2005; Tenchov et al., 2006; Xu et al., 2007; Angelini et al., 2012; Oger and Cario, 2013; Meador et al., 2014; Kellermann et al., 2016). During culturing experiments with neutrophilic *H. volcanii*, a correlation between the amount of PGP-Me and the Mg^2+^ concentration was observed (Kellermann et al., 2016) and was hypothesized to relate to the closely positioned, two negatively charged phosphate moieties which allow binding to divalent cation to reduce the permeability of monovalent cations under high salt concentrations [cf. Figure 9 of Kellermann et al. (2016)]. As stated above, six of the nine *Halobacteria* strains examined were haloalkaliphiles, while three were haloneutrophiles and all nine were cultured at very low Mg^2+^ concentrations (1 mM). Kellermann et al. (2016) suggested that in order to maintain membrane stability at low Mg^2+^ concentrations, haloneutrophiles adapt by upregulating cardiolipins in place of PG or PGP-Me, while haloalkaliphiles utilize the combined EXT-AR and PGP-Me approach described above. However, in our study we did not see a difference between the neutrophilic and alkaliphiles haloarchaea in terms of the relative amounts of cardiolipin, PG and PGP-Me or EXT-AR. Therefore, it is the Mg^2+^ concentration which is more important in this case, rather than the pH. It should be noted however that at high pH only low levels of Mg^2+^ remain in solution.

#### Characteristic Lipids of the Methanomicrobia

While the two *Methanonatronarchaeia* and *Mc. alkaliphilus* AMF2^T^ all exhibited relatively common diether core lipid compositions (AR and Uns-AR), *Ms. natronophilum* AME2^T^ had the most complex diether lipid composition of any of the thirteen strains: based on GC-MS analysis (Table 2) it contained 45% AR, 15% OH-AR, and 6% MAR. The 16% of alkyl glycerol monoethers represents the Uns-AR and Uns-OH-AR identified in intact form by LC-ITMS (Tables S1, S5). The presence of OH-AR alongside AR is considered characteristic of the *Methanosarcinales* order to which *Ms. natronophilum* AME2^T^ belongs (Koga et al., 1998). The hydroxylation of AR to produce OH-AR has been postulated to have an effect on membrane stability, possibly increasing the polar surface area of the membrane or by creating hydrophilic pockets (Sprott et al., 1990). While MAR is not considered to be a general characteristic of the *Methanosarcinales* order, it has been previously reported in certain genera of the order, such as extremely halophilic *Methanohalobium* (Koga et al., 1998). The presence of MAR phospholipids has been shown to improve membrane impermeability to protons and solute leakage and membrane stability in methanogenic archaea (Koga et al., 1998) in a similar way to tetraether lipids. This structural effect is thought to explain its accumulation in the hyperthermophilic methanogen *Methanococcus jannaschii* [where it can account for up to 85% of the core lipids; (Sprott et al., 1991; Dannenmuller et al., 2000)], and other members of the *Methanococcales* order (Koga et al., 1998). The same might be true for extremely halophilic methanogens. As discussed above, *Ms. natronophilum* AME2^T^ is an extremely salt-tolerant alkaliphile (cultured at 3 M total Na^+^) and the MAR and OH-AR core lipid adaptions of this strain may be order- or species-specific adaptions to high salt and high pH conditions.

Two closely related polar headgroups (tri, di, and mono N-methyl), APT and APT-Me were detected only in the single *Methanomicrobiales* member *Mc. alkaliphilus* AMF2^*T*^. While (tri, di- and mono) N-methyl APT-Me are all novel compounds, trimethyl APT has been described in a range of methanogenic euryarchaea, including the members of the order *Methanomicrobiales*, but, in agreement with our findings, not in the order *Methanosarcinales* (Ferrante et al., 1987; Koga et al., 1993, 1998; Koga and Morii, 2005). While APT has been considered previously a characteristic lipid headgroup of methanogenic archaea (Koga et al., 1993), our finding suggests it may be specific at the order level. It would be expected that the zwitterionic nature of these two headgroups would mean that they provide an overall neutral charge at the membrane surface and hence would not bind to cations in the same manner as negatively charged (anionic) lipids, e.g., PGP-Me (cf. section Characteristic lipids of the *Halobacteria*). This predominantly neutral membrane surface may have evolved to adapt to the conditions encountered by *Mc. alkaliphilus* AMF2^T^ of moderate salt (as per the culturing conditions 0.6 M Na^+^, 1 mM Mg^2+^) and high pH (9.5–10). It is of note that Szekely et al. (2011) found that, when modeling interactions between lipid membranes and various ions, while divalent ions do bind to zwitterionic headgroups, the presence of at least one double bond in the lipid tail is sufficient to prevent this divalent ion adsorption. The authors suggested that this was due to the looser packing of unsaturated lipids, allowing free rotation of the headgroup. It is of note therefore that *Mc. alkaliphilus* AMF2^T^, contained a high amount of unsaturated diether lipids of all 13 strains examined (12% alkyl glycerol monoethers formed during acid hydrolysis from unsaturated diether lipids, Table 2). This suggests that the presence of high levels of zwitterionic headgroups and of unsaturated lipids in AMF2^T^ are interrelated membrane adaptions.

It should be noted that the other member of the *Methanomicrobia* class, *Ms. natronophilum* AME2^T^, also contained a high proportion of Uns-ARs (16%, Table 2), whereas in all other classes it was <5%. Dawson et al. (2012) studied the effect of salinity on AR saturation in four strains of haloarchaea and found that the degree of unsaturation increased with increasing salinity of growth medium as well as with the strains' increasing salinity optimum. They hypothesized that increased AR unsaturation is a physiological adaptation to both maintain membrane fluidity and reduce ion-permeability as salinity increases. Kellermann et al. (2016) observed for *H. volcanii* an increase in the degree of unsaturation with increasing Na^+^ levels primarily within the bulk IPL PG-AR (although they noted that total AR did not exhibit the same trend in unsaturation, which they suggested was due to impaired growth performance at both the low and high ends of the salt concentration range). However, a higher salt concentration does not explain the high level of AR unsaturation in the two *Methanomicrobia* as both *Ms. natronophilum* AME2^T^ and *Mc. alkaliphilus* AMF2^T^ were cultured at the lowest salt concentrations of all 13 strains (2 M and 0.6 M total Na^+^). This suggests that AR unsaturation in response to salt concentrations is a class specific adaption, manifested at lower salt concentrations by these *Methanomicrobia* than the *Methanonatronarchaeia* and *Halobacteria*, which are all more extreme halophiles.

#### Lipids of the Natrialbales

A group of lipids only seen in members of the order *Natrialbales* included three PGPGP cardiolipins, one with two AR cores, one with an AR and an EXT-AR core and the final one with two EXT-AR cores (Table S1). These PGPGP cardiolipins were present in alkaliphilic polysaccharide utilizing haloarchaea “*Na*. *chitinivorans*” AArcht4^T^, *Nb. cellulositropha* AArcel5^T^ and AArcel7, but not in the less alkaliphilic “*Ha. desulfuricum”* AArc-Sl^T^. These PGPGP cardiolipins may be the same as unknown cardiolipins, postulated to be glycocardiolipins, detected previously in two species of alkaliphilic haloarchaea from the order *Halobacteriales* (Angelini et al., 2012). If this is the case then PGPGP cardiolipins may be characteristic of the alkaliphilic members of *Halobacteria* growing at low Mg background. It has been previously suggested, based on results from culturing experiments with the neutrophilic *Halobacteriales* member *H. volcanii* that at low Mg^2+^ concentration, the membrane upregulates the amount of PGP cardiolipin, to assure membrane integrity [cf. Figure 9 of Kellermann et al. (2016)]. At high Mg^2+^ levels, *H. volcanii* upregulated PGP-Me to increase Mg^2+^ binding at its closely positioned phosphate moieties (Kellermann et al., 2016). Thus, it may be possible that PGPGP headgroups could be orientated in a way that neighboring phosphate moieties are tethered via divalent cations, as per PGP-Me-AR, while also benefiting from the additional structural integrity of the cardiolipin structure.

The polar headgroup assigned as PG-Gly was only detected in two alkaliphilic *Natrialbales* (*Nb. cellulositropha* AArcel5 and AArcel7) but in no other orders. The PG-Gly headgroup with a diacyl core has been described in halophilic gammaproteobacteria of the genus *Halomonas* (Giordano et al., 2007), where it was postulated that the bulky headgroup would enhance steric protection through hydrogen bonding via glycosyl headgroups to ensure the osmotic stability of the cellular membrane under high-salt conditions. Furthermore, a very similar phosphoglycolipid with a sulfated sugar moiety was identified in alkaliphilic haloarchaeon *N. moolapensis* from the *Halobacteriales* order (Hoffmann et al., 2015). The presence of this headgroup in both extremely halophilic archaea and moderately halophilic bacteria further supports the hypothesis that this headgroup provides additional osmotic stability.

#### Neutrophilic Strains

Cellulolytic “*Hc. cellulosivorans”* HArcel1^T^ and sulfur-respiring *Hd. formicicum* HSR6^T^ and *Hn. sulfurireducens* HSR2^T^ of the order *Halobacteriales* all contained the PGS headgroup, not seen in any of the other 10 strains as well as the novel sulfur-containing headgroup (**IIIa-d**), which was also seen in methanogenic “*Ca*. Mh. thermophilum” HMET1. The presence of these sulfur containing lipids may be a distinctive characteristic associated with these *Halobacteriales* members or may relate to the neutrophilic nature of these extreme halophiles. Indeed, while nine strains out of 13 examined were alkaliphilic, the remaining four neutrophilic strains (cf. Table 1): methanogenic '*Ca*. Mh. thermophilum' HMET1, sulfur-respiring *Hd. formicicum* HSR6^T^ and *Hn. sulfurireducens* HSR2^T^ and aerobic polysaccharide utilizing “*Hc. cellulosivorans”* HArcel1^T^ were all cultured at pH 7. PGS was only detected in three of these strains (not in HMET1) and not in any of the alkaliphilic strains (Table 3). Indeed, PGS has been reported as a characteristic component in the other neutrophilic haloarchaea, such as the members of *Haloferax* and *Halorubrum lacusprofundi* from the *Haloferacales* (Kates, 1992; Gibson et al., 2005) and *Halomicrobium mukohataei* from the *Halobacteriales* (Oren et al., 2002).

Another characteristic of the neutrophilic strains was the presence of glycolipids. All four neutrophilic strains contained the 2Gly polar headgroup with an AR core, which was only detected in trace levels in two alkaliphiles, *Mc. alkaliphilus* AMF2^T^ and *Nb. cellulositropha* AArcel5^T^ (Table 3). Similarly, the lipid composition of “*Ca*. Mh. thermophilum” HMET1 was dominated by 2Gly-GDGT-0 whereas the three alkaliphilic methanogens only produced GDGTs with phospho-based polar headgroups (Tables 3, S1). This supports the assertion that glycolipids are absent or present only in trace amounts in membranes of alkaliphilic representatives of extremely halophilic Euryarchaeota (Oger and Cario, 2013; Siliakus et al., 2017).

### Biosynthesis of EXT-AR

Low levels of an IPL cardiolipin with an EXT-AR core in '*Ca*. Mh. thermophilum' HMET1 (Table S1; supported by the presence of EXT-AR in hydrolyzed core lipids, Table 2), is an interesting result as, of the 8 known orders of methanogens, only a representative of *Methanomassiliicoccales* has been described to date as containing EXT-AR (Koga and Morii, 2005; Becker et al., 2016). Indeed, the analyzed strains in the class *Methanomicrobia* (*Ms. natronophilum* AME2^T^ and *Mc. alkaliphilus* AMF2^T^) did not contain any EXT-AR, whereas all analyzed *Halobacteria* did (cf. Tables 2, S5). These results led us to the hypothesis that the close relationship between the novel class-level lineage *Methanonatronarchaeia* and the class *Halobacteria* (Sorokin et al., 2017a, cf. Figure 1) means that they share the same genetic capability to produce EXT-AR. It should be noted, however, that EXT-AR was only present in low levels in “*Ca*. Mh. thermophilum” HMET1 (Tables 2, S1) and that *Mn. thermophilum* AMET1^T^, also a member of the *Methanonatronarchaeia*, did not contain any EXT-AR. It is possible that the presence of EXT-AR in HMET1 and not AMET1^T^ is related to culture conditions as the medium for HMET1 contained 4 M NaCl, while that of AMET1^T^ contained 2 M Na^+^ as NaCl and 2 M Na^+^ as carbonates. Hence the osmotic pressure in case of AMET1^T^ was much lower, since sodium carbonates, as weak electrolytes, impose roughly two time less osmotic pressure than the strongly electrolytic NaCl (Robinson and Macaskill, 1979). Dawson et al. (2012) noted previously that, while the presence of EXT-AR seems to be in part taxonomic, an increase in its abundance is advantageous for haloarchaea as it decreases membrane ion-permeability. As described above, the presence of EXT-AR has been suggested to provide a “zipper” like effect between the two layers of the membrane providing increased membrane stability (De Rosa et al., 1982). It has also been found that C_25_-C_25_ EXT-AR liposomes are thicker than C_20_-C_20_ AR liposomes and have relatively lower permeability (Gmajner et al., 2011), so a similar effect could be expected of the C_20_-C_25_ EXT-AR detected in this study.

**Figure 9:**
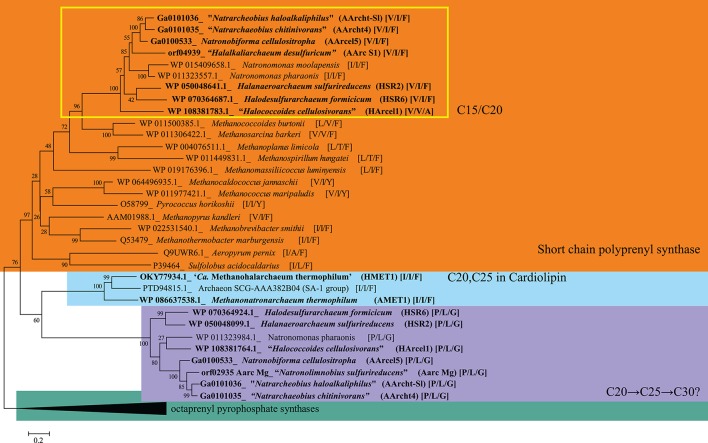
Phylogenetic tree of putative polyprenyl synthases involved in isoprenoid elongation. Phylogenetic reconstruction was performed by maximum likelihood as indicated in the methods. The percentage of replicate trees in which the associated taxa clustered together in the bootstrap test (1,000 replicates) are shown next to the branches. The tree is drawn to scale, with branch lengths in the same units as those of the evolutionary distances used to infer the phylogenetic tree. The nature of the amino acids at the 11th, 8th, and 5th position before the aspartate-rich motif is given in square brackets e.g., (AA_11_, AA_8_, AA_5_). Full strain names given in Table 1. Amino acid codes: V, valine; I, isoleucine; F, phenylalanine; A, alanine; L, leucine; T, threonine; Y, tyrosine; P, proline; G, glycine.

In order to examine the genetic capability to produce EXT-AR we examined the polyprenyl synthases (belonging to the prenyltransferase family) involved in the elongation of isoprenoids (Liang et al., 2002) in the genomes of the two members of *Methanonatronarchaeia* class along with eight haloarchaeal isolates from this study plus additional archaeal species, including a single-cell genome sequence, from Nereus Deep in the Red Sea, belonging to a putatively novel family within the archaeal candidate division SA1 (Ngugi and Stingl, 2018), to which the two members of *Methanonatronarchaeia* also belong (Figure 9). Homology search analysis revealed multiple putative polyprenyl synthases in the analyzed genomes. Specific amino acid (AA) residues of the polyprenyl synthase genes are known to determine the length of the isoprenoid chain. Specifically, the fifth amino acid before the First Aspartate-Rich Motif (FARM) has been found to determine the length of the formed isoprenoid chain. For the case of the C_25_ isoprenoid chains of the EXT-AR, also the eighth and 11th amino acids are important in the determination of the C_25_ extension (Liang et al., 2002). Figure 9 illustrates the relationships between the different polyprenyl synthases present in the archaeal genomes examined and the nature of the amino acids at the 11th, 8th, and 5th position before the aspartate-rich motif (given in square brackets e.g., [AA_11_, AA_8_, AA_5_]). Bulky amino acids such as phenylalanine (F) block the length of the isoprenoids at C_20_, while smaller amino acids such as serine (S) allow for elongation to C_25_ and beyond (Ohnuma et al., 1996; Liang et al., 2002).

Many of the examined archaeal genomes encode multiple putative polyprenyl synthases, generally separated in function by the nature of the specific FARM amino acids described above (Figure 9). A major cluster of synthases, which we assigned as long chain polyprenyl synthases (Figure 9), are all characterized by a P/L/G (proline_11_, leucine_8_, glycine_5_) FARM motif. Indeed, replacement of bulky F at the fifth position with a smaller amino acid residue such as serine (S) has been described as the most effective way to elongate beyond a C_20_ chain (Ohnuma et al., 1996; Liang et al., 2002). A similar effect would be expected from G at the fifth position, as it is smaller still than serine. The second major cluster, which we assigned as short chain polyprenyl synthases, was characterized by the presence of motives with the bulky, chain-limiting F at the fifth position. It should be noted here that the polyprenyl synthases clustering does not reflect a dichotomy between species that have been shown to produce or not produce EXT-AR. Indeed, many species contain both the short and long chain polyprenyl synthases, as would be expected as they produce a range of isoprenoid-based products with varying chain lengths. Another point to note is that *A. pernix* is known to produce exclusively a C_25_, C_25_ AR (Morii et al., 1999). However, it only contained one putative polyprenyl synthase, which clustered with the short chain polyprenyl synthases (Figure 9), most closely with acidophilic *S. acidocaldarius* which does not produce EXT-AR (Sprott et al., 1997). In this case it is important to note that it is the FARM motif of *A. pernix* I/A/F (isoleucine_11_, alanine_8_, F_5_) that distinguishes the enzymes' ability to form extended isoprenoids beyond the C_20_ length. Indeed, in the case of *A. pernix* it is the presence of the relatively small and compact A at the eighth position which potentially determines the C_25_ chain length of the product (Liang et al., 2002). Conversely, *S. acidocaldarius* contains the bulkier L in the eighth position. The clustering of these genes together, despite this difference, suggests that the polyprenyl synthase of *A. pernix* was originally a short chain polyprenyl synthase which underwent certain amino acid replacements, but overall remains most closely related to the other short chain polyprenyl synthases. Interestingly, although the methanogen *Methanomassiliicoccus luminyensis* has been described as containing EXT-AR (Becker et al., 2016), based on its very bulky, chain-limiting FARM motif (L_11_, I_8_, F_5_) as pointed out in Figure 9, this would not be expected.

A third cluster of polyprenyl synthases contained the three members of the archaeal candidate division SA1: the only two members of *Methanonatronarchaeia* presently known in culture, “*Ca*. Mh. thermophilum” HMET1 and *Mn. thermophilum* AMET1^T^ and a single-cell genome sequence from Nereus Deep in the Red Sea. All three only contained one putative polyprenyl synthase with two Is, and the relatively bulky F at the 11th, 8th, and 5th positions, a motif associated with short chain polyprenyl synthase. However, these putative polyprenyl synthases cluster far apart from all the other short chain polyprenyl synthases and cluster closer to the long chain polyprenyl synthases (Figure 9). While this conflicting motif and clustering provide no specific evidence that this is the gene involved in the production of EXT-AR containing cardiolipins in *Methanonatronarchaeia*, it does raise the possibility that these novel extremophilic methanogens utilize a different isoprenoid elongation mechanism than the other extremely halophilic euryarchaea examined here. Perhaps their isolated phylogenetic position has afforded them alternative pathways to adapt their membranes to hypersaline conditions using EXT-AR. However, as EXT-AR was only present in low levels in '*Ca*. Mh. thermophilum' HMET1 (Tables 2, S1) and was not present in *Mn. thermophilum* AMET1^T^, it does not appear to be a significant membrane adaptation under the culturing conditions examined in this study.

## Conclusions

Analyzing a large set of novel halo(alkali)philic representatives of Euryarchaeota, novel diether-based lipids were detected including a PG-Gly headgroup combined with a diether core, an APT-like lipid with an additional methoxy group in place of a hydroxy group on the pentanetetrol and finally headgroups with elemental composition of either C_12_H_25_NO_13_S or C_12_H_25_NO_16_S_2_. Furthermore, novel cardiolipins were detected in three strains: a putative PGPGP polar group bridging the diether components. The presence of cardiolipins with EXT-AR in one of the *Methanonatronarchaeia* strains was unusual, as only one other methanogenic archaeal order has been reported to produce EXT-AR. Both *Methanonatronarchaeia* strains contain a specific polyprenyl synthase which clustered separately from those of the haloarchaea. This may indicate the potential of these strains to synthesize EXT-AR.

We compared the distribution of membrane lipids across the 13 strains to examine what factors determined lipid composition, including the phylogenetic relationships between the strains, the three different metabolic processes they carryout and adaptions associated with the pH. In general we found that the lipid distribution of the 13 strains could be generally separated into two, the methanogens (group) and the *Halobacteria* (class) based on the presence of specific core lipids. Within the methanogen group adaption to high or more moderate salt concentration resulted in the extremely halophilic class *Methanonatronarchaeia* containing more GDGTs than AR, while the less halophilic members of the class *Methanomicrobia* contained more AR than GDGTs. The methanogen *Ms. natronophilum* AME2^T^ had the most complex diether lipid composition of any of the 13 strains, including OH-AR and MAR which we surmise is an order-specific membrane adaption. The zwitterionic lipids APT and APT-Me headgroups were detected only in the *Methanomicrobiales* member *Mc. alkaliphilus* AMF2^T^, which also contained the highest proportion of unsaturated lipids. Many of the *Halobacteria* strains contained a high proportion of the EXT-AR core and PGP-Me headgroup in agreement with the other cultured genera from the class. Within the *Halobacteria* only alkaliphilic members of the *Natrialbales* order contained PGPGP cardiolipins and the PG-Gly headgroup. The four neutrophilic strains belonging to *Haloferacales* and *Halobacteriales* orders were characterized by the presence of sulfur-containing headgroups and glycolipids.

## Data Availability

All datasets generated for this study are included in the manuscript and/or the supplementary files.

## Author Contributions

NB carried out data analysis and wrote manuscript. DS isolated and cultured archaea. EH oversaw analytical methods and carried out data analysis. MK and WR carried out extractions and data analysis. LV carried out homology search and phylogenetic analyses. HW carried out NMR analysis and JS conceived and supervised the project.

### Conflict of Interest Statement

The authors declare that the research was conducted in the absence of any commercial or financial relationships that could be construed as a potential conflict of interest.

## References

[B1] Amoozegar M. A. Siroosi M. Atashgahi S. Smidt H. Ventosa A. (2017). Systematics of haloarchaea and biotechnological potential of their hydrolytic enzymes. Microbiology 163, 623–645. 10.1099/mic.0.000463 28548036

[B2] Andrei A.-S. Banciu H. L. Oren A. (2012). Living with salt: metabolic and phylogenetic diversity of archaea inhabiting saline ecosystems. FEMS Microbiol. Lett. 330, 1–9. 10.1111/j.1574-6968.2012.02526.x 22339687

[B3] Angelini R. Corral P. Lopalco P. Ventosa A. Corcelli A. (2012). Novel ether lipid cardiolipins in archaeal membranes of extreme haloalkaliphiles. Biochim. Biophys. Acta 1818, 1365–1373. 10.1016/j.bbamem.2012.02.014 22366205

[B4] Becker K. W. Elling F. J. Yoshinaga M. Y. Söllinger A. Urich T. Hinrichs K.-U. (2016). Unusual butane- and pentanetriol-based tetraether lipids in *Methanomassiliicoccus luminyensis*, a representative of the seventh order of methanogens. Appl. Environ. Microbiol. 82, 4505–4516. 10.1128/AEM.00772-16 27208108 PMC4984300

[B5] Begemann M. B. Mormile M. R. Paul V. G. Vidt D. J. (2011). Potential enhancement of biofuel production through enzymatic biomass degradation activity and biodiesel production by halophilic microorganisms in Halophiles and Hypersaline Environments: Current Research and Future Trends, eds. Ventosa A. Oren A. Ma Y. (Berlin; Heidelberg: Springer Berlin Heidelberg), 341–357. 10.1007/978-3-642-20198-1_18

[B6] Besseling M. A. Hopmans E. C. Boschman R. C. Damsté S. S J. Villanueva L. (2018). Benthic archaea as potential sources of tetraether membrane lipids in sediments across an oxygen minimum zone. Biogeosciences 15, 4047–4064. 10.5194/bg-15-4047-2018

[B7] Corcelli A. (2009). The cardiolipin analogues of Archaea. Biochim. Biophys. Acta 1788, 2101–2106. 10.1016/j.bbamem.2009.05.010 19464258

[B8] Corcelli A. Colella M. Mascolo G. Fanizzi F. P. Kates M. (2000). A novel glycolipid and phospholipid in the purple membrane. Biochemistry 39, 3318–3326. 10.1021/bi992462z 10727224

[B9] Dannenmuller O. Arakawa K. Eguchi T. Kakinuma K. Blanc S. Albrecht A.-M.. (2000). Membrane properties of archæal macrocyclic diether phospholipids. Chemistry 6, 645–654. 10.1002/(SICI)1521-3765(20000218)6:4<645::AID-CHEM645>3.0.CO;2-A;2-A 10807176

[B10] Dawson K. S. Freeman K. H. Macalady J. L. (2012). Molecular characterization of core lipids from halophilic archaea grown under different salinity conditions. Org. Geochem. 48, 1–8. 10.1016/j.orggeochem.2012.04.003

[B11] De Rosa M. Gambacorta A. Nicolaus B. Ross H. N. M. Grant W. D. Bu'Lock J. D. (1982). An asymmetric archaebacterial diether lipid from alkaliphilic halophiles. Microbiology 128, 343–348. 10.1099/00221287-128-2-343

[B12] de Souza L. M. Müller-Santos M. Iacomini M. Gorin P. A. J. Sassaki G. L. (2009). Positive and negative tandem mass spectrometric fingerprints of lipids from the halophilic Archaea *Haloarcula marismortui*. J. Lipid Res. 50, 1363–1373. 10.1194/jlr.M800478-JLR200 19258281 PMC2694335

[B13] Edgar R. C. (2004). MUSCLE: multiple sequence alignment with high accuracy and high throughput. Nucleic Acids Res. 32, 1792–1797. 10.1093/nar/gkh340 15034147 PMC390337

[B14] Elling F. J. Könneke M. Mußmann M. Greve A. Hinrichs K.-U. (2015). Influence of temperature, pH, and salinity on membrane lipid composition and TEX86 of marine planktonic thaumarchaeal isolates. Geochim. Cosmochim. Acta 171, 238–255. 10.1016/j.gca.2015.09.004

[B15] Ferrante G. Ekiel I. Sprott G. D. (1987). Structures of diether lipids of *Methanospirillum hungatei* containing novel head groups *N*,*N*-diniethylamino- and *N*,*N*,*N*-trimethylaminopentanetetrol. Biochim. Biophys. Acta 921, 281–291. 10.1016/0005-2760(87)90029-4 7918605

[B16] Gibson J. A. E. Miller M. R. Davies N. W. Neill G. P. Nichols D. S. Volkman J. K. (2005). Unsaturated diether lipids in the psychrotrophic archaeon *Halorubrum lacusprofundi*. Syst. Appl. Microbiol. 28, 19–26. 10.1016/j.syapm.2004.09.004 15709361

[B17] Gibson R. A. van der Meer M. T. J. Hopmans E. C. Reysenbach A.-L. Schouten S. Sinninghe Damsté J. S. (2013). Comparison of intact polar lipid with microbial community composition of vent deposits of the Rainbow and Lucky Strike hydrothermal fields. Geobiology 11, 72–85. 10.1111/gbi.12017 23231657

[B18] Giordano A. Vella F. M. Romano I. Gambacorta A. (2007). Structural elucidation of a novel phosphoglycolipid isolated from six species of *Halomonas*. J. Lipid Res. 48, 1825–1831. 10.1194/jlr.M700152-JLR200 17519342

[B19] Gmajner D. Ota A. Šentjurc M. Ulrih N. P. (2011). Stability of diether C25,25 liposomes from the hyperthermophilic archaeon *Aeropyrum pernix* K1. Chem. Phys. Lipids 164, 236–245. 10.1016/j.chemphyslip.2011.01.005 21295560

[B20] Hoffmann A. Kovermann M. Oberwinkler T. Siedler F. Cortina N. S. Balbach J.. (2015). Novel sulfated phosphoglycolipids from Natronomonas moolapensis. Chem. Phys. Lipids 191, 8–15. 10.1016/j.chemphyslip.2015.06.004 26134137

[B21] Hopmans E. C. Schouten S. Pancost R. D. van der Meer M. T. Sinninghe Damsté J. S. (2000). Analysis of intact tetraether lipids in archaeal cell material and sediments by high performance liquid chromatography/atmospheric pressure chemical ionization mass spectrometry. Rapid Commun. Mass Spectrom. 14, 585–589. 10.1002/(SICI)1097-0231(20000415)14:7<585::AID-RCM913>3.0.CO;2-N 10775092

[B22] Jones D. T. Taylor W. R. Thornton J. M. (1992). The rapid generation of mutation data matrices from protein sequences. Comput. Appl. Biosci. 8, 275–282. 1633570 10.1093/bioinformatics/8.3.275

[B23] Jukes T. H. Cantor C. R. (1969). Evolution of protein molecules in Mammalian Protein Metabolism, III. eds Munro H. N. (New York, NY: Academic Press), 21–132.

[B24] Kates M. (1992). Archaebacterial lipids: structure, biosynthesis and function in The Archaebacteria: Biochemistry and Biotechnology, eds. Danson M. J. Hough D. W. Lunt G. G. (London: Portland Press), 51–72. 1445410

[B25] Kates M. Moldoveanu N. Stewart L. C. (1993). On the revised structure of the major phospholipid of *Halobacterium salinarium*. Biochim. Biophys. Acta 1169, 46–53. 8334149 10.1016/0005-2760(93)90080-s

[B26] Kellermann M. Y. Yoshinaga M. Y. Valentine R. C. Wörmer L. Valentine D. L. (2016). Important roles for membrane lipids in haloarchaeal bioenergetics. Biochim. Biophys. Acta 1858, 2940–2956. 10.1016/j.bbamem.2016.08.010 27565574

[B27] Knittel K. Boetius A. (2009). Anaerobic oxidation of methane: progress with an unknown process. Annu. Rev. Microbiol. 63, 311–334. 10.1146/annurev.micro.61.080706.093130 19575572

[B28] Koga Y. Morii H. (2005). Recent advances in structural research on ether lipids from archaea including comparative and physiological aspects. Biosci. Biotechnol. Biochem. 69, 2019–2034. 10.1271/bbb.69.2019 16306681

[B29] Koga Y. Morii H. Akagawa-Matsushita M. Ohga M. (1998). Correlation of polar lipid composition with 16S rRNA phylogeny in methanogens. Further Analysis of Lipid Component Parts. Biosci. Biotechnol. Biochem. 62, 230–236. 10.1271/bbb.62.230 27388514

[B30] Koga Y. Nakano M. (2008). A dendrogram of archaea based on lipid component parts composition and its relationship to rRNA phylogeny. Syst. Appl. Microbiol. 31, 169–182. 10.1016/j.syapm.2008.02.005 18515030

[B31] Koga Y. Nishihara M. Morii H. Akagawa-Matsushita M. (1993). Ether polar lipids of methanogenic bacteria: structures, comparative aspects, and biosyntheses. Microbiol. Rev. 57, 164–182. 8464404 10.1128/mr.57.1.164-182.1993 PMC372904

[B32] Kushwaha S. C. Kates M. Sprott G. D. Smith I. C. (1981). Novel complex polar lipids from the methanogenic archaebacterium *Methanospirillum hungatei*. Science 211, 1163–1164. 10.1126/science.7466385 7466385

[B33] Kushwaha S. C. Kramer J. K. G. Kates M. (1975). Isolation and characterization of C50-carotenoid pigments and other polar isoprenoids from *Halobacterium cutirubrum*. Biochim. Biophys. Acta 398, 303–314. 10.1016/0005-2760(75)90146-0 1182141

[B34] Lattanzio V. M. Corcelli A. Mascolo G. Oren A. (2002). Presence of two novel cardiolipins in the halophilic archaeal community in the crystallizer brines from the salterns of Margherita di Savoia (Italy) and Eilat (Israel). Extremophiles 6, 437–444. 10.1007/s00792-002-0279-2 12486451

[B35] Liang P.-H. Ko T.-P. Wang A. H.-J. (2002). Structure, mechanism and function of prenyltransferases. Eur. J. Biochem. 269, 3339–3354. 10.1046/j.1432-1033.2002.03014.x 12135472

[B36] Lipp J. S. Hinrichs K.-U. (2009). Structural diversity and fate of intact polar lipids in marine sediments. Geochim. Cosmochim. Acta 73, 6816–6833. 10.1016/j.gca.2009.08.003

[B37] Lipp J. S. Morono Y. Inagaki F. Hinrichs K.-U. (2008). Significant contribution of Archaea to extant biomass in marine subsurface sediments. Nature 454, 991–994. 10.1038/nature07174 18641632

[B38] Liu X.-L. Leider A. Gillespie A. Gröger J. Versteegh G. J. M. Hinrichs K.-U. (2010). Identification of polar lipid precursors of the ubiquitous branched GDGT orphan lipids in a peat bog in Northern Germany. Org. Geochem. 41, 653–660. 10.1016/j.orggeochem.2010.04.004

[B39] Lobasso S. Pérez-Davó A. Vitale R. Sánchez M. M.-, Corcelli, A. (2015). Deciphering archaeal glycolipids of an extremely halophilic archaeon of the genus *Halobellus* by MALDI-TOF/MS. Chem. Phys. Lipids 186, 1–8. 10.1016/j.chemphyslip.2014.11.002 25447292

[B40] Mathai J. C. Sprott G. D. Zeidel M. L. (2001). Molecular mechanisms of water and solute transport across archaebacterial lipid membranes. J. Biol. Chem. 276, 27266–27271. 10.1074/jbc.M103265200 11373291

[B41] Meador T. B. Gagen E. J. Loscar M. E. Goldhammer T. Yoshinaga M. Y. Wendt J.. (2014). *Thermococcus kodakarensis* modulates its polar membrane lipids and elemental composition according to growth stage and phosphate availability. Front. Microbiol. 5:10. 10.3389/fmicb.2014.00010 24523718 PMC3906577

[B42] Minegishi H. Echigo A. Nagaoka S. Kamekura M. Usami R. (2010). Halarchaeum acidiphilum gen. nov., sp. nov., a moderately acidophilic haloarchaeon isolated from commercial solar salt. Int. J. Syst. Evol. Microbiol. 60, 2513–2516. 10.1099/ijs.0.013722-0 19965997

[B43] Morii H. Yagi H. Akutsu H. Nomura N. Sako Y. Koga Y. (1999). A novel phosphoglycolipid archaetidyl(glucosyl)inositol with two sesterterpanyl chains from the aerobic hyperthermophilic archaeon *Aeropyrum pernix* K1. Biochim. Biophys. Acta 1436, 426–436. 9989273 10.1016/s0005-2760(98)00157-x

[B44] Ngugi D. K. Stingl U. (2018). High-quality draft single-cell genome sequence belonging to the archaeal candidate division SA1, isolated from nereus deep in the Red Sea. Genome Announc. 6, e00383–e00318. 10.1128/genomeA.00383-18 29748404 PMC5946040

[B45] Nichols D. S. Miller M. R. Davies N. W. Goodchild A. Raftery M. Cavicchioli R. (2004). Cold adaptation in the Antarctic Archaeon *Methanococcoides burtonii* involves membrane lipid unsaturation. J. Bacteriol. 186, 8508–8515. 10.1128/JB.186.24.8508-8515.2004 15576801 PMC532414

[B46] Nishihara M. Koga Y. (1991). Hydroxyarchaetidylserine and hydroxyarchaetidyl-myo-inositol in *Methanosarcina barkeri*: polar lipids with a new ether core portion. Biochim. Biophys. Acta 1082, 211–217 1901027 10.1016/0005-2760(91)90196-o

[B47] Oger P. M. Cario A. (2013). Adaptation of the membrane in Archaea. Biophys. Chem. 183, 42–56. 10.1016/j.bpc.2013.06.020 23915818

[B48] Ohnuma S. Hirooka K. Hemmi H. Ishida C. Ohto C. Nishino T. (1996). Conversion of product specificity of Archaebacterial Geranylgeranyl-diphosphate Synthase Identification of essential amino acid residues for chain length determination of prenyltransferase reaction. J. Biol. Chem. 271, 18831–18837. 10.1074/jbc.271.31.18831 8702542

[B49] Oren A. (1999). Bioenergetic aspects of halophilism. Microbiol. Mol. Biol. Rev. 63, 334–348. 10357854 10.1128/mmbr.63.2.334-348.1999 PMC98969

[B50] Oren A. (2002). Molecular ecology of extremely halophilic Archaea and Bacteria. FEMS Microbiol. Ecol. 39, 1–7. 10.1111/j.1574-6941.2002.tb00900.x 19709178

[B51] Oren A. Elevi R. Watanabe S. Ihara K. Corcelli A. (2002). Halomicrobium mukohataei gen. nov., comb. nov., and emended description of Halomicrobium mukohataei. Int. J. Syst. Evol. Microbiol. 52, 1831–1835. 10.1099/00207713-52-5-1831 12361294

[B52] Pancost R. D. Bouloubassi I. Aloisi G. Sinninghe Damsté J. S. Scientific Party, the M. S (2001). Three series of non-isoprenoidal dialkyl glycerol diethers in cold-seep carbonate crusts. Org. Geochem. 32, 695–707. 10.1016/S0146-6380(01)00015-8

[B53] Pitcher A. Hopmans E. C. Mosier A. C. Park S.-J. Rhee S.-K. Francis C. A.. (2011). Core and intact polar glycerol dibiphytanyl glycerol tetraether lipids of ammonia-oxidizing archaea enriched from marine and estuarine sediments. Appl. Environ. Microbiol. 77, 3468–3477. 10.1128/AEM.02758-10 21441324 PMC3126447

[B54] Robinson R. A. Macaskill J. B. (1979). Osmotic coefficients of aqueous sodium carbonate solutions at 25°C. J. Solution Chem. 8, 35–40. 10.1007/BF00646807

[B55] Rossel Cartes P. (2009). Microbial Communities Performing Anaerobic Oxidation of Methane: Diversity of Lipid Signatures and Habitats. University of Bremen.

[B56] Rossel P. Lipp J. Fredricks H. Arnds J. Boetius A. Elvert M. (2008). Intact polar lipids of anaerobic methanotrophic Archaea and associated bacteria. Org. Geochem. 39, 992–999. 10.1016/j.orggeochem.2008.02.021

[B57] Rossel P. E. Elvert M. Ramette A. Boetius A. Hinrichs K.-U. (2011). Factors controlling the distribution of anaerobic methanotrophic communities in marine environments: evidence from intact polar membrane lipids. Geochim. Cosmochim. Acta 75, 164–184. 10.1016/j.gca.2010.09.031

[B58] Saitou N. Nei M. (1987). The neighbor-joining method: a new method for reconstructing phylogenetic trees. Mol. Biol. Evol. 4, 406–425. 10.1093/oxfordjournals.molbev.a040454 3447015

[B59] Schouten S. Hopmans E. C. Baas M. Boumann H. Standfest S. Koenneke M. (2008). Intact membrane lipids of “*Candidatus* Nitrosopumilus maritimus,” a cultivated representative of the cosmopolitan mesophilic group I crenarchaeota. Appl. Environ. Microbiol. 74, 2433–2440. 10.1128/AEM.01709-07 18296531 PMC2293165

[B60] Schouten S. Hopmans E. C. Sinninghe Damsté J. S. (2013). The organic geochemistry of glycerol dialkyl glycerol tetraether lipids: a review. Org. Geochem. 54, 19–61. 10.1016/j.orggeochem.2012.09.006

[B61] Schubotz F. Wakeham S. G. Lipp J. S. Fredricks H. F. Hinrichs K.-U. (2009). Detection of microbial biomass by intact polar membrane lipid analysis in the water column and surface sediments of the Black Sea. Environ. Microbiol. 11, 2720–2734. 10.1111/j.1462-2920.2009.01999.x 19624710

[B62] Shinoda W. Shinoda K. Baba T. Mikami M. (2005). Molecular dynamics study of bipolar tetraether lipid membranes. Biophys. J. 89, 3195–3202. 10.1529/biophysj.105.060962 16100279 PMC1366815

[B63] Siliakus M. F. van der Oost J. Kengen S. W. M. (2017). Adaptations of archaeal and bacterial membranes to variations in temperature, pH and pressure. Extremophiles 21, 651–670. 10.1007/s00792-017-0939-x 28508135 PMC5487899

[B64] Sinninghe Damsté J. S. Rijpstra W. I. C. Hopmans E. C. Weijers J. W. H. Foesel B. U. Overmann J. (2011). 13,16-Dimethyl Octacosanedioic Acid (iso-Diabolic Acid), a common membrane-spanning lipid of acidobacteria subdivisions 1 and 3. Appl. Environ. Microbiol. 77, 4147–4154. 10.1128/AEM.00466-11 21515715 PMC3131667

[B65] Sinninghe Damsté J. S. Schouten S. Hopmans E. C. van Duin A. C. T. Geenevasen J. A. J. (2002). Crenarchaeol: the characteristic core glycerol dibiphytanyl glycerol tetraether membrane lipid of cosmopolitan pelagic crenarchaeota. J. Lipid Res. 43, 1641–1651. 10.1194/jlr.M200148-JLR200 12364548

[B66] Sorokin D. Y. Abbas B. Geleijnse M. Pimenov N. V. Sukhacheva M. V. van Loosdrecht M. C. M. (2015a). Methanogenesis at extremely haloalkaline conditions in the soda lakes of Kulunda Steppe (Altai, Russia). FEMS Microbiol. Ecol. 91:fiv016. 10.1093/femsec/fiv016 25764464

[B67] Sorokin D. Y. Abbas B. Merkel A. Y. Rijpstra W. I. C. Damsté J. S. S. Sukhacheva M. V.. (2015b). *Methanosalsum natronophilum* sp. nov., and Methanocalculus alkaliphilus sp. nov., haloalkaliphilic methanogens from hypersaline soda lakes. Int. J. Syst. Evol. Microbiol. 65, 3739–3745. 10.1099/ijsem.0.000488 26228570

[B68] Sorokin D. Y. Elcheninov A. G. Toshchakov S. V. Bale N. J. Sinninghe Damsté J. S. Khijniak T. V.. (2019). *Natrarchaeobius chitinivorans* gen. nov., sp. nov., and *Natrarchaeobius halalkaliphilus* sp. nov., alkaliphilic, chitin-utilizing haloarchaea from hypersaline alkaline lakes. Syst. Appl. Microbiol. [Epub ahead of print]. 10.1016/j.syapm.2019.01.001. 30638904 PMC6542413

[B69] Sorokin D. Y. Khijniak T. V. Kostrikina N. A. Elcheninov A. G. Toshchakov S. V. Bale N. J.. (2018a). *Natronobiforma cellulositropha* gen. nov., sp. nov., a novel haloalkaliphilic member of the family Natrialbaceae (class Halobacteria) from hypersaline alkaline lakes. Syst. Appl. Microbiol. 41, 355–362. 10.1016/j.syapm.2018.04.002 29752017 PMC6052348

[B70] Sorokin D. Y. Kublanov I. V. Gavrilov S. N. Rojo D. Roman P. Golyshin P. N.. (2016a). Elemental sulfur and acetate can support life of a novel strictly anaerobic haloarchaeon. ISME J. 10, 240–252. 10.1038/ismej.2015.79 25978546 PMC4681856

[B71] Sorokin D. Y. Kublanov I. V. Yakimov M. M. Rijpstra W. I. C. Sinninghe Damsté J. S. (2016b). *Halanaeroarchaeum sulfurireducens* gen. nov., sp. nov., the first obligately anaerobic sulfur-respiring haloarchaeon, isolated from a hypersaline lake. Int. J. Syst. Evol. Microbiol. 66, 2377–2381. 10.1099/ijsem.0.001041 27031647

[B72] Sorokin D. Y. Makarova K. S. Abbas B. Ferrer M. Golyshin P. N. Galinski E. A.. (2017a). Discovery of extremely halophilic, methyl-reducing euryarchaea provides insights into the evolutionary origin of methanogenesis. Nat. Microbiol. 2:17081. 10.1038/nmicrobiol.2017.81 28555626 PMC5494993

[B73] Sorokin D. Y. Merkel A. Y. Abbas B. Makarova K. S. Rijpstra W. I. C. Koenen M. (2018b). *Methanonatronarchaeum thermophilum* gen. nov., sp. nov. and “*Candidatus* Methanohalarchaeum thermophilum”, extremely halo(natrono)philic methyl-reducing methanogens from hypersaline lakes comprising a new euryarchaeal class *Methanonatronarchaeia classis* nov. Int. J. Syst. Evol. Microbiol. 68, 2199–2208. 10.1099/ijsem.0.002810 29781801 PMC6978985

[B74] Sorokin D. Y. Messina E. La Cono V. Ferrer M. Ciordia S. Mena M. C.. (2018c). Sulfur Respiration in a group of facultatively anaerobic natronoarchaea ubiquitous in hypersaline soda lakes. Front. Microbiol. 9:2359. 10.3389/fmicb.2018.02359 30333814 PMC6176080

[B75] Sorokin D. Y. Messina E. Smedile F. Roman P. Damsté J. S. S. Ciordia S.. (2017b). Discovery of anaerobic lithoheterotrophic haloarchaea, ubiquitous in hypersaline habitats. ISME J. 11, 1245–1260. 10.1038/ismej.2016.203 28106880 PMC5437934

[B76] Sorokin D. Y. Toshchakov S. V. Kolganova T. V. Kublanov I. V. (2015c). Halo(natrono)archaea isolated from hypersaline lakes utilize cellulose and chitin as growth substrates. Front. Microbiol. 6:942. 10.3389/fmicb.2015.00942 26441877 PMC4569967

[B77] Sprott G. D. Agnew B. J. Patel G. B. (1997). Structural features of ether lipids in the archaeobacterial thermophiles *Pyrococcus furiosus, Methanopyrus kandleri, Methanothermus fervidus*, and *Sulfolobus acidocaldarius*. Can. J. Microbiol. 43, 467–476. 10.1139/m97-066

[B78] Sprott G. D. Ekiel I. Dicaire C. (1990). Novel, acid-labile, hydroxydiether lipid cores in methanogenic bacteria. J. Biol. Chem. 265, 13735–13740. 2380184

[B79] Sprott G. D. Meloche M. Richards J. C. (1991). Proportions of diether, macrocyclic diether, and tetraether lipids in *Methanococcus jannaschii* grown at different temperatures. J. Bacteriol. 173, 3907–3910. 2050642 10.1128/jb.173.12.3907-3910.1991 PMC208025

[B80] Sturt H. F. Summons R. E. Smith K. Elvert M. Hinrichs K. U. (2004). Intact polar membrane lipids in prokaryotes and sediments deciphered by high-performance liquid chromatography/electrospray ionization multistage mass spectrometry - new biomarkers for biogeochemistry and microbial ecology. Rapid Commun. Mass Spectrom. 18, 617–628. 10.1002/rcm.1378 15052572

[B81] Szekely O. Steiner A. Szekely P. Amit E. Asor R. Tamburu C.. (2011). The structure of ions and zwitterionic lipids regulates the charge of dipolar membranes. Langmuir 27, 7419–7438. 10.1021/la200264s 21598965

[B82] Tachibana A. (1994). A novel prenyltransferase, farnesylgeranyl diphosphate synthase, from the haloalkaliphilic archaeon, *Natronobacterium pharaonis*. FEBS Lett. 341, 291–294. 8137956 10.1016/0014-5793(94)80475-3

[B83] Tachibana A. Yano Y. Otani S. Nomura N. Sako Y. Taniguchi M. (2000). Novel prenyltransferase gene encoding farnesylgeranyl diphosphate synthase from a hyperthermophilic archaeon, *Aeropyrum pernix*. Molecularevolution with alteration in product specificity. Eur. J. Biochem. 267, 321–328. 10.1046/j.1432-1327.2000.00967.x 10632701

[B84] Tamura K. Stecher G. Peterson D. Filipski A. Kumar S. (2013). MEGA6: molecular evolutionary genetics analysis version 6.0. Mol. Biol. Evol. 30, 2725–2729. 10.1093/molbev/mst197 24132122 PMC3840312

[B85] Teixidor P. Grimait J. O. Pueyo J. J. Rodriguez-Valera F. (1993). Isopranylglycerol diethers in non-alkaline evaporitic environments. Geochim. Cosmochim. Acta 57, 4479–4489. 10.1016/0016-7037(93)90497-K

[B86] Tenchov B. Vescio E. M. Sprott G. D. Zeidel M. L. Mathai J. C. (2006). Salt tolerance of archaeal extremely halophilic lipid membranes. J. Biol. Chem. 281, 10016–10023. 10.1074/jbc.M600369200 16484230

[B87] Tindall B. J. Ross H. N. M. Grant W. D. (1984). Natronobacterium gen. nov. and Natronococcus gen. nov., two new genera of haloalkaliphilic archaebacteria. Syst. Appl. Microbiol. 5, 41–57. 10.1016/S0723-2020(84)80050-8

[B88] Valentine D. L. (2007). Adaptations to energy stress dictate the ecology and evolution of the Archaea. Nat. Rev. Microbiol. 5, 316–323. 10.1038/nrmicro1619 17334387

[B89] Villanueva L. Damsté J. S. S. Schouten S. (2014). A re-evaluation of the archaeal membrane lipid biosynthetic pathway. Nat. Rev. Microbiol. 12, 438–448. 10.1038/nrmicro3260 24801941

[B90] Wang Q. Li W. Yang H. Liu Y. Cao H. Dornmayr-Pfaffenhuemer M.. (2007). *Halococcus qingdaonensis* sp. nov., a halophilic archaeon isolated from a crude sea-salt sample. Int. J. Syst. Evol. Microbiol. 57, 600–604. 10.1099/ijs.0.64673-0 17329792 PMC3182530

[B91] Xu X.-W. Wu Y.-H. Wang C.-S. Oren A. Zhou P.-J. Wu M. (2007). *Haloferax larsenii* sp. nov., an extremely halophilic archaeon from a solar saltern. Int. J. Syst. Evol. Microbiol. 57, 717–720. 10.1099/ijs.0.64573-0 17392193

[B92] Xu Y. Wang Z. Xue Y. Zhou P. Ma Y. Ventosa A.. (2001). *Natrialba hulunbeirensis* sp. nov. and Natrialba chahannaoensis sp. nov., novel haloalkaliphilic archaea from soda lakes in Inner Mongolia Autonomous Region, China. Int. J. Syst. Evol. Microbiol. 51, 1693–1698. 10.1099/00207713-51-5-1693 11594597

[B93] Xu Y. Zhou P. Tian X. (1999). Characterization of two novel haloalkaliphilic archaea *Natronorubrum bangense* gen. nov., sp. nov. and *Natronorubrum tibetense* gen. nov., sp. nov. Int. J. Syst. Bacteriol. 49(Pt 1), 261–266. 10.1099/00207713-49-1-261 10028271

[B94] Xue Y. Fan H. Ventosa A. Grant W. D. Jones B. E. Cowan D. A.. (2005). *Halalkalicoccus tibetensis* gen. nov., sp. nov., representing a novel genus of haloalkaliphilic archaea. Int. J. Syst. Evol. Microbiol. 55, 2501–2505. 10.1099/ijs.0.63916-0 16280517

[B95] Yoshinaga M. Y. Kellermann M. Y. Rossel P. E. Schubotz F. Lipp J. S. Hinrichs K.-U. (2011). Systematic fragmentation patterns of archaeal intact polar lipids by high-performance liquid chromatography/electrospray ionization ion-trap mass spectrometry. Rapid Commun. Mass Spectrom. 25, 3563–3574. 10.1002/rcm.5251 22095505

[B96] Yoshinaga M. Y. Wörmer L. Elvert M. Hinrichs K.-U. (2012). Novel cardiolipins from uncultured methane-metabolizing archaea. Archaea 2012:832097. 10.1155/2012/832097 22654563 PMC3359654

