# Supplementary material for: New Insights Into the Polar Lipid Composition of Extremely Halo(alkali)philic Euryarchaea From Hypersaline Lakes
Source: Front Microbiol. 2019 Mar 12;10:377. doi: 10.3389/fmicb.2019.00377 (PMC6423904; doi:10.3389/fmicb.2019.00377)
Supplement: Supplementary file 1 [file Table_1.docx]

**Supplement to:**

**New insights into the polar lipid composition of** **extremely halo(alkali)philic euryarchaea from hypersaline lakes**

**Nicole J. Bale^1*^, Dimitry Y. Sorokin^2,3^, Ellen C. Hopmans^1^, Michel Koenen^1^, W. Irene C. Rijpstra^1^, Laura Villanueva^1^, Hans Wienk^4^, Jaap S. Sinninghe Damsté^1,5^**

^1^NIOZ Royal Institute for Sea Research, Department of Marine Microbiology and Biogeochemistry, and Utrecht University, P.O. Box 59, 1790 AB Den Burg, Texel, The Netherlands

^2^Winogradsky Institute of Microbiology, Research Centre of Biotechnology, Russian Academy of Sciences, Moscow, Russia

*^3^Delft University of Technology, Faculty of Applied Sciences, Department of Biotechnology, van der Maasweg 9, 2629 HZ, Delft, The Netherlands*

^4^Utrecht University, NMR Spectroscopy, Bijvoet Center for Biomolecular Research, Utrecht, The Netherlands

^5^Utrecht University, Faculty of Geosciences, Department of Earth Sciences, P.O. Box 80.121, 3508 TA, Utrecht, The Netherlands.

**Correspondence:**

**Dr Nicole Bale**

[**nicole.bale@nioz.nl**](mailto:nicole.bale@nioz.nl)

**Table Legends:**

**Table S1. IPLs identified by HPLC-ITMS and their abundance (in percent of total ion current) in haloarchaea isolated from hypersaline lakes.**

**Table S2. Accurate masses of APT-AR and novel lipids IIa, IIb, IIc and their fragments (UHPLC-HRMS MS^2^).**

**Table S3. Selected ^13^C- and ^1^H-NMR signals (ppm) from N,N,N-trimethyl aminopentanetetrol (APT) and methoxy-APT (APT-Me). AR = archaeol.**

**Table S4. Accurate masses of novel lipids IIIa-d and their fragments (UHPLC-HRMS MS^2^).**

**Table S5. Distribution of core lipids in diethers and cardiolipins from HPLC-ITMS analysis. IPLs were examined in terms of their peak area response and thus the proportions reported do not reflect quantitative relative abundance and hence the data is represented qualitatively. See Table 2 for quantitative analysis. See text for abbreviations.**

**Table S1. IPLs identified by HPLC-ITMS and their relative abundance (in percent of total peak area) in haloarchaea isolated from hypersaline lakes.**

|  |  |  |  |  | **Methanogens** | | | | **Anaerobic sulfur reducers** | | | | **Polysaccharide utilizers**  **Cellulolytics Chitinolytics** | | | | |
| --- | --- | --- | --- | --- | --- | --- | --- | --- | --- | --- | --- | --- | --- | --- | --- | --- | --- |
| ***m/z*** | **Adduct type** | **Head group** | **Core lipid** | **Diagnostic fragments^a^** | AMET1^T^ | HMET1 | AME2^T^ | AMF2^T^ | HSR2^T^ | HSR6^T^ | AArc-Mg | AArc-Sl^T^ | HArcel1^T^ | AArcel5 | AArcel7 | AArcht4^T^ | AArcht-Sl^T^ |
| **Diethers** | |  |  |  |  |  |  |  |  |  |  |  |  |  |  |  |  |
| 776.8 | [M+H]^+^ | PE | AR | 733, 453, 357 | 1 |  | 1 |  | 2 | 3 |  |  |  |  |  | 1 |  |
| 807.8 | [M+H]^+^ | PG | AR | 733, 653, 527, 373 | 6 | 8 | 2 | 3 | 5 | 6 | 10 | 6 | 4 | 7 | 4 | 9 | 9 |
| 805.2 | [M+H]^+^ |  | Uns(1)-AR | 787, 731, 651, 527/525, 509, 371, 335 | 1 | 2 | 1 | 0.1 | 1 | 0.1 | 1 | 1 | 1 | 1 | 0.3 | 1 | 1 |
| 820.1 | [M+NH_4_]^+^ |  | Uns(2)-AR | 527, 247 | 2 | 4 | 2 | 0.2 | 1 | 0.1 | 1 | 3 | 2 | 2 | 1 | 4 | 1 |
| 818.2 | [M+NH_4_]^+^ |  | Uns(3)-AR | 522, 247 | 2 | 3 | 2 | 0.3 | 1 | 0.1 | 0.3 | 2 | 2 | 1 | 0.2 | 1 | 1 |
| 816.2 | [M+NH_4_]^+^ |  | Uns(4)-AR | 522, 247 | 2 | 2 | 2 | 0.2 | 1 | <0.1 | 0.4 | 3 | 2 | 1 | 0.4 | 1 | 1 |
| 814.3 | [M+NH_4_]^+^ |  | Uns(5)-AR | 520, 247 | 3 | 1 | 3 | 0.2 | 1 | 0.1 | 0.3 | 2 | 1 | 1 | 1 | 0.2 | 0.5 |
| 812.2 | [M+NH_4_]^+^ |  | Uns(6)-AR | 519, 247 | 2 | 1 | 9 | 0.2 | 1 | 0.1 | 1 | 3 | 2 | 2 | 1 | 0.2 | 1 |
| 810.2 | [M+NH_4_]^+^ |  | Uns(7)-AR | 519, 247 | 3 | 3 | 2 | 0.2 | 0.1 | <0.1 |  |  | 0.2 | 0.3 |  |  |  |
| 808.3 | [M+NH_4_]^+^ |  | Uns(8)-AR | 518, 247 | 8 | 17 | 1 | 0.4 |  |  |  |  | 0.3 | 0.3 |  |  |  |
| 877.8 | [M+H]^+^ |  | EXT-AR | 803, 723, 527, 373 |  |  |  |  | 4 | 5 | 8 | 2 | 1 | 5 | 8 | 8 | 9 |
| 892.2 | [M+NH_4_]^+^ |  | Uns(1)-EXT-AR | 597, 527, 247 |  |  |  |  | 0.4 | 0.1 | 1 | 1 | 0.4 | 1 | 2 | 1 | 0.3 |
| 890.3 | [M+NH_4_]^+^ |  | Uns(2)-EXT-AR | 597, 549, 247 |  |  |  |  | 0.3 | 0.1 | 2 | 1 | 1 | 0.3 | 2 | 1 | 0.3 |
| 888.3 | [M+NH_4_]^+^ |  | Uns(3)-EXT-AR | 547, 269, 247 |  |  |  |  | 0.3 | <0.1 | 3 | 2 | 1 | 1 | 1 | 1 | 0.3 |
| 886.3 | [M+NH_4_]^+^ |  | Uns(4)-EXT-AR | 524, 247 |  |  |  |  | 1 | <0.1 | 1 | 2 | 3 | 1 | 1 | 1 | 0.5 |
| 884.2 | [M+NH_4_]^+^ |  | Uns(5)-EXT-AR | 543, 524, 269, 247 |  |  |  |  | 1 | 0.1 | 1 | 0.3 | 2 | 2 | 7 | 2 | 1 |
| 882.2 | [M+NH_4_]^+^ |  | Uns(6)-EXT-AR | 520, 247 |  |  |  |  | 1 | 0.2 | 2 | 1 | 2 | 1 | 3 | 1 | 1 |
| 880.3 | [M+NH_4_]^+^ |  | Uns(7)-EXT-AR | 541, 269, 247 |  |  |  |  | 2 |  | 2 | 1 | 4 | 3 | 10 | 2 | 2 |
| 878.3 | [M+NH_4_]^+^ |  | Uns(8)-EXT-AR | 247 |  |  |  |  | 1 |  | 0.5 | 0.1 | 1 | 1 | 1 |  | 0.5 |
| 805.8 | [M+H]^+^ |  | MAR | 731,651,615 |  |  | 0.5 |  |  |  |  |  |  |  |  |  |  |
| 904.8 | [M+NH_4_]^+^ | PGP | AR | 887.68 | 0.2 | 5 | 1 |  | 1 | 0.2 | 1 | 1 | 1 | 1 | 2 | 0.4 | 3 |
| 923.0 | [M+H]^+^ | N,N,N-trimethyl APT-Me | AR | 642, 270, 208 |  |  |  | 21 |  |  |  |  |  |  |  |  |  |
| 920.9 | [M+H]^+^ |  | Uns(1)-AR | 642, 270, 208 |  |  |  | 2 |  |  |  |  |  |  |  |  |  |
| 918.9 | [M+H]^+^ |  | Uns(2)-AR | 642, 208 |  |  |  | 6 |  |  |  |  |  |  |  |  |  |
| 916.9 | [M+H]^+^ |  | Uns(3)-AR | 642, 362, 208 |  |  |  | 15 |  |  |  |  |  |  |  |  |  |
| 914.9 | [M+H]^+^ |  | Uns(4)-AR | 642/640/638/636, 362, 208 |  |  |  | 3 |  |  |  |  |  |  |  |  |  |
| 912.8 | [M+H]^+^ |  | Uns(5)-AR | 638/636, 362, 208 |  |  |  | 4 |  |  |  |  |  |  |  |  |  |
| 910.9 | [M+H]^+^ |  | Uns(6)-AR | 636, 362, 208 |  |  |  | 4 |  |  |  |  |  |  |  |  |  |
| 908.9 | [M+H]^+^ |  | Uns(7)-AR | 636/634, 362, 208 |  |  |  | 1 |  |  |  |  |  |  |  |  |  |
| 906.9 | [M+H]^+^ |  | Uns(8)-AR | 634, 362, 208 |  |  |  | 0.5 |  |  |  |  |  |  |  |  |  |
| 908.9 | [M+H]^+^ | N,N-dimethyl APT-Me | AR | 628, 610, 274, 256, 194 |  |  |  | 1 |  |  |  |  |  |  |  |  |  |
| 906.8 | [M+H]^+^ |  | Uns(1)-AR | na |  |  |  | 0.1 |  |  |  |  |  |  |  |  |  |
| 904.9 | [M+H]^+^ |  | Uns(2)-AR | 628, 194 |  |  |  | 0.3 |  |  |  |  |  |  |  |  |  |
| 902.8 | [M+H]^+^ |  | Uns(3)-AR | 628, 348, 194 |  |  |  | 0.1 |  |  |  |  |  |  |  |  |  |
| 900.7 | [M+H]^+^ |  | Uns(4)-AR | na |  |  |  | <0.1 |  |  |  |  |  |  |  |  |  |
| 898.7 | [M+H]^+^ |  | Uns(5)-AR | na |  |  |  | <0.1 |  |  |  |  |  |  |  |  |  |
| 896.7 | [M+H]^+^ |  | Uns(6)-AR | na |  |  |  | <0.1 |  |  |  |  |  |  |  |  |  |
| 894.7 | [M+H]^+^ | N-monomethyl APT-Me | AR | na |  |  |  | <0.1 |  |  |  |  |  |  |  |  |  |
| 890.8 | [M+H]^+^ |  | Uns(2)-AR | na |  |  |  | <0.1 |  |  |  |  |  |  |  |  |  |
| 888.7 | [M+H]^+^ |  | Uns(3)-AR | na |  |  |  | <0.1 |  |  |  |  |  |  |  |  |  |
| 909.0 | [M+H]^+^ | N,N,N-trimethyl APT | AR | 628, 256, 194 |  |  |  | 9 |  |  |  |  |  |  |  |  |  |
| 907.0 | [M+H]^+^ |  | Uns(1)-AR | 628, 256, 194 |  |  |  | 1 |  |  |  |  |  |  |  |  |  |
| 904.9 | [M+H]^+^ |  | Uns(2)-AR | 628, 194 |  |  |  | 2 |  |  |  |  |  |  |  |  |  |
| 902.8 | [M+H]^+^ |  | Uns(3)-AR | 628, 348, 194 |  |  |  | 5 |  |  |  |  |  |  |  |  |  |
| 900.8 | [M+H]^+^ |  | Uns(4)-AR | 626, 348, 194 |  |  |  | 2 |  |  |  |  |  |  |  |  |  |
| 898.8 | [M+H]^+^ |  | Uns(5)-AR | 622, 348, 194 |  |  |  | 2 |  |  |  |  |  |  |  |  |  |
| 896.8 | [M+H]^+^ |  | Uns(6)-AR | 622, 348, 194 |  |  |  | 1 |  |  |  |  |  |  |  |  |  |
| 894.8 | [M+H]^+^ |  | Uns(7)-AR | 622/620, 348, 194 |  |  |  | 0.4 |  |  |  |  |  |  |  |  |  |
| 894.8 | [M+H]^+^ | N,N-dimethyl APT | AR | 614, 260, 180 |  |  |  | 0.1 |  |  |  |  |  |  |  |  |  |
| 892.7 | [M+H]^+^ |  | Uns(1)-AR | na |  |  |  | <0.1 |  |  |  |  |  |  |  |  |  |
| 890.7 | [M+H]^+^ |  | Uns(2)-AR | na |  |  |  | <0.1 |  |  |  |  |  |  |  |  |  |
| 888.7 | [M+H]^+^ |  | Uns(3)-AR | na |  |  |  | <0.1 |  |  |  |  |  |  |  |  |  |
| 886.7 | [M+H]^+^ |  | Uns(4)-AR | na |  |  |  | <0.1 |  |  |  |  |  |  |  |  |  |
| 884.6 | [M+H]^+^ |  | Uns(5)-AR | na |  |  |  | <0.1 |  |  |  |  |  |  |  |  |  |
| 882.8 | [M+H]^+^ |  | Uns(6)-AR | na |  |  |  | <0.1 |  |  |  |  |  |  |  |  |  |
| 880.9 | [M+H]^+^ | N-monomethyl APT | AR | 733, 600, 228 |  |  |  | 0.1 |  |  |  |  |  |  |  |  |  |
| 878.8 | [M+H]^+^ |  | Uns(1)-AR | na |  |  |  | <0.1 |  |  |  |  |  |  |  |  |  |
| 876.7 | [M+H]^+^ |  | Uns(2)-AR | na |  |  |  | <0.1 |  |  |  |  |  |  |  |  |  |
| 874.7 | [M+H]^+^ |  | Uns(3)-AR | na |  |  |  | <0.1 |  |  |  |  |  |  |  |  |  |
| 895.8 | [M+H]^+^ | PI | AR | 733, 653, 615, 299 |  |  | 1 |  |  |  |  |  |  |  |  |  |  |
| 911.7 | [M+H]^+^ |  | OH-AR | 893, 615, 261 |  |  | 14 |  |  |  |  |  |  |  |  |  |  |
| 909.5 | [M+H]^+^ |  | Uns(1)-OH-AR | 631, 613, 335 |  |  | 2 |  |  |  |  |  |  |  |  |  |  |
| 907.4 | [M+H]^+^ |  | Uns(2)-OH-AR | 629, 613, 335 |  |  | 1 |  |  |  |  |  |  |  |  |  |  |
| 922.6 | [M+NH_4_]^+^ |  | Uns(3)-OH-AR | 647, 357 |  |  | 1 |  |  |  |  |  |  |  |  |  |  |
| 920.3 | [M+NH_4_]^+^ |  | Uns(4)-OH-AR | 645, 357, 335 |  |  | 3 |  |  |  |  |  |  |  |  |  |  |
| 918.5 | [M+NH_4_]^+^ |  | Uns(5)-OH-AR | 643, 357, 335 |  |  | 15 |  |  |  |  |  |  |  |  |  |  |
| 820.7 | [M+H]^+^ | PS | AR | 733, 540, 357 | 3 |  | 1 |  |  |  |  |  |  |  |  |  |  |
| 818.4 | [M+H]^+^ |  | Uns(1)-AR | 731, 540 | 1 |  | 0.2 |  |  |  |  |  |  |  |  |  |  |
| 816.4 | [M+H]^+^ |  | Uns(2)-AR | 538, 260 | 0.5 |  | 0.2 |  |  |  |  |  |  |  |  |  |  |
| 814.5 | [M+H]^+^ |  | Uns(3)-AR | 538, 260 | 0.3 |  | 0.2 |  |  |  |  |  |  |  |  |  |  |
| 812.4 | [M+H]^+^ |  | Uns(4)-AR | 536, 260 | 0.3 |  | 0.1 |  |  |  |  |  |  |  |  |  |  |
| 810.4 | [M+H]^+^ |  | Uns(5)-AR | 534, 260 | 1 |  | 0.5 |  |  |  |  |  |  |  |  |  |  |
| 808.5 | [M+H]^+^ |  | Uns(6)-AR | 532, 260 | 1 |  | 3 |  |  |  |  |  |  |  |  |  |  |
| 806.4 | [M+H]^+^ |  | Uns(7)-AR | 532, 260 | 2 |  | 0.4 |  |  |  |  |  |  |  |  |  |  |
| 974.6 | [M+NH_4_]^+^ | PGS | EXT-AR | 249 |  |  |  |  | 5 | 11 |  |  | <0.1 |  |  |  |  |
| 887.7 | [M+H]^+^ |  | AR | 807 |  |  |  |  | 3 | 11 |  |  | 4 |  |  |  |  |
| 971.8 | [M+H]^+^ | PGP-Me | EXT-AR | 953, 939, 249 |  |  |  |  | 25 | 26 | 23 | 10 | 12 | 16 | 26 | 23 | 24 |
| 969.8 | [M+H]^+^ |  | Uns(1)-EXT-AR | 951, 937, 249 |  |  |  |  |  |  | 4 | 4 | 3 | 3 |  |  | 2 |
| 967.7 | [M+H]^+^ |  | Uns(2)-EXT-AR | 949, 935, 249 |  |  |  |  |  |  | 2 | 9 | 1 |  |  |  | 6 |
| 965.8 | [M+H]^+^ |  | Uns(3)-EXT-AR | 947, 933, 249 |  |  |  |  |  |  | 10 | 8 | 4 | 1 |  |  | 1 |
| 901.8 | [M+H]^+^ |  | AR | 883, 869, 249 | 30 | 3 | 30 |  | 21 | 23 | 28 | 37 | 31 | 27 | 22 | 34 | 35 |
| 1039.7 | [M+H]^+^ | PG-Gly | EXT-AR | 877 |  |  |  |  |  |  |  |  |  | 8 | 4 |  |  |
| 969.7 | [M+H]^+^ |  | AR | 807 |  |  |  |  |  |  |  |  |  | 12 |  |  |  |
| 1064.8 | [M+NH_4_]^+^ | 2Gly | EXT-AR | 723 |  |  |  |  | 4 | 0.1 |  |  | 0.3 | 2 |  |  |  |
| 994.7 | [M+NH_4_]^+^ |  | AR | 653 |  | 29 |  | 2 | 1 |  |  |  | 0.3 | 1 |  |  |  |
| 1241.4 | [M+NH_4_]^+^ | (C_12_H_22_NO_10_(SO_3_H)_2_) | EXT-AR | 1142, 406 |  |  |  |  | <0.1 | 3 |  |  |  |  |  |  |  |
| 1171.4 | [M+NH_4_]^+^ |  | AR | 1074, 994, 406 |  |  |  |  | 0.2 | 8 |  |  |  |  |  |  |  |
| 1161.5 | [M+NH_4_]^+^ | (C_12_H_22_NO_10_(SO_3_H)) | EXT-AR | 1064, |  |  |  |  | 9 | 1 |  |  | 5 |  |  |  |  |
| 1091.5 | [M+NH_4_]^+^ |  | AR | 1074, 994 |  | 9 |  |  | 8 | 2 |  |  | 11 |  |  |  |  |
| **Cardiolipins** | |  |  |  |  |  |  |  |  |  |  |  |  |  |  |  |  |
| 1522.3 | [M+H]^+^ | PGP (BPG) | AR, AR | 1504, 869, 789 |  | 4 |  |  |  |  |  |  |  |  |  |  |  |
| 1592.4 | [M+H]^+^ |  | AR, EXT-AR | 1574, 939, 869 |  | 2 |  |  |  |  |  |  |  |  |  |  |  |
| 1676.4 | [M+H]^+^ | PGPGP | AR, AR | 1396, 1023, 869, 807 |  |  |  |  |  |  |  |  |  | 1 | 0.2 | 3 |  |
| 1746.5 | [M+H]^+^ |  | AR, EXT-AR | 1466, 1093, 1023, 939, 869, 807 |  |  |  |  |  |  |  |  |  | 0.3 | 1 | 4 |  |
| 1816.6 | [M+H]^+^ |  | EXT-AR, EXT-AR | 1536, 1093, 939, 877 |  |  |  |  |  |  |  |  |  |  | 4 | 2 |  |
| **GDGTS** | |  |  |  |  |  |  |  |  |  |  |  |  |  |  |  |  |
| 1643.1 | [M+NH_4_]^+^ | 2Gly | GDGT-0 | 1302 |  | 8 |  |  |  |  |  |  |  |  |  |  |  |
| 1610.6 | [M+H]^+^ | PG--PG | GDGT-0 | 1592, 1456, 1438 | 24 |  | 1 | 11 |  |  |  |  |  |  |  |  |  |
| 1608.6 | [M+H]^+^ |  | GDGT-1 | 1590, 1454, 1436 | 6 |  | 0.4 | 1 |  |  |  |  |  |  |  |  |  |
| 1579.5 | [M+H]^+^ | PG--PE | GDGT-0 | 1425, 1382, 776 | 2 |  |  |  |  |  |  |  |  |  |  |  |  |

na = Compound identification based on retention time (in relation to known compounds) as the MS^2^ spectrum was not available (due to low response). *m/z* values are observed not calculated. PE = phosphatidylethanolamine, PS = phosphatidylserine, PI = phosphatidylinositol, PG = phosphatidylglycerol, PGP = phosphatidylglycerophosphate, PGP-Me = phosphatidylglycerophosphate methyl ester, PGS = phosphatidylglycerosulfate, APT = aminopentanetetrol, APT-Me = aminomethoxypentanetriol, PG-Gly = phosphatidylglycerohexose, 2Gly = dihexose. PGPGP = phosphatidylglycerophosphate glycerophosphate, BPG = bisphosphatidylglycerol, GDGT = glycerol dialkyl glycerol tetraether (where n is the number of cyclopentane moieties). AR = archaeol, EXT-AR = extended archaeol, MAR = macrocyclic archaeol, OH-AR = hydroxy archaeol. Full strain names given in Table 1.

**Table S2. Accurate masses of APT-AR and novel lipids IIa, IIb, IIc and their fragments (UHPLC-HRMS MS^2^).**

|  | **[M+H]^+^** | **Assigned elemental**  **composition of [M+H]^+^** | **Δ mmu** | **MS^2^ product ions** | **Assigned elemental**  **composition of MS^2^ product ions** | **Δ mmu** |
| --- | --- | --- | --- | --- | --- | --- |
| N,N,N-trimethyl APT-AR | 908.7666 | C_51_H_107_NO_9_P | -1.2 | 176.1284  194.1399  256.0953  274.1037 | C_8_H_18_NO_3_  C_8_H_20_NO_4_  C_8_H_19_NO_6_P  C_8_H_21_NO_7_P | 0.3  1.2  0.8  -1.3 |
| N,N-dimethyl APT-AR | 894.7510 | C_50_H_105_NO_9_P | -1.1 | 162.1125  180.1230  260.0894 | C_7_H_16_NO_3_  C_7_H_18_NO_4_  C_7_H_19_NO_7_P | 0.0  0.0  0.0 |
| N-monomethyl APT-AR | 880.7349 | C_49_H_103_NO_9_P | -1.6 | 148.0969  166.1079  228.0634 | C_6_H_14_NO_3_  C_6_H_16_NO_4_  C_6_H_15_NO_6_P | 0.1  0.5  0.2 |
| IIa | 922.7816 | C_52_H_109_NO_9_P | -1.8 | 190.1438  208.1544  270.1100  288.1205 | C_9_H_20_NO_3_  C_9_H_22_NO_4_  C_9_H_21_NO_6_P  C_9_H_23_NO_7_P | 0.0  0.1  -0.1  -0.2 |
| IIb | 908.7661 | C_51_H_107_NO_9_P | -1.7 | 176.1282  194.1388  274.1049 | C_8_H_18_NO_3_  C_8_H_20_NO_4_  C_8_H_21_NO_7_P | 0.1  0.1  -0.1 |
| IIc | 894.7506 | C_50_H_105_NO_9_P | -1.5 | 162.1124  180.1230  260.0894 | C_7_H_16_NO_3_  C_7_H_18_NO_4_  C_7_H_19_NO_7_P | -0.1  0.0  0.0 |

mmu = milli mass unit, **Δ mmu** = (measured mass – calculated mass) x 1000, APT = aminopentanetetrol, APT-Me = aminomethoxypentanetriol, AR = archaeol.

**Table S3. Selected ^13^C- and ^1^H-NMR signals (ppm) from N,N,N-trimethyl APT and N,N,N-trimethyl APT-Me.**

|  |  | 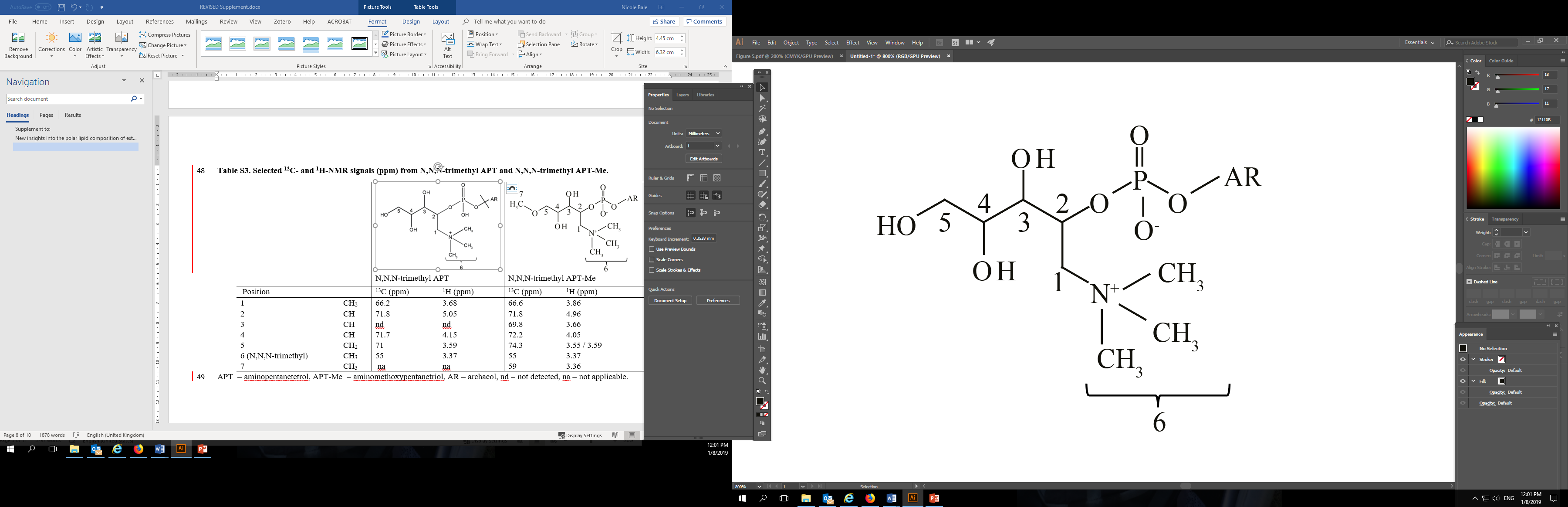 | | 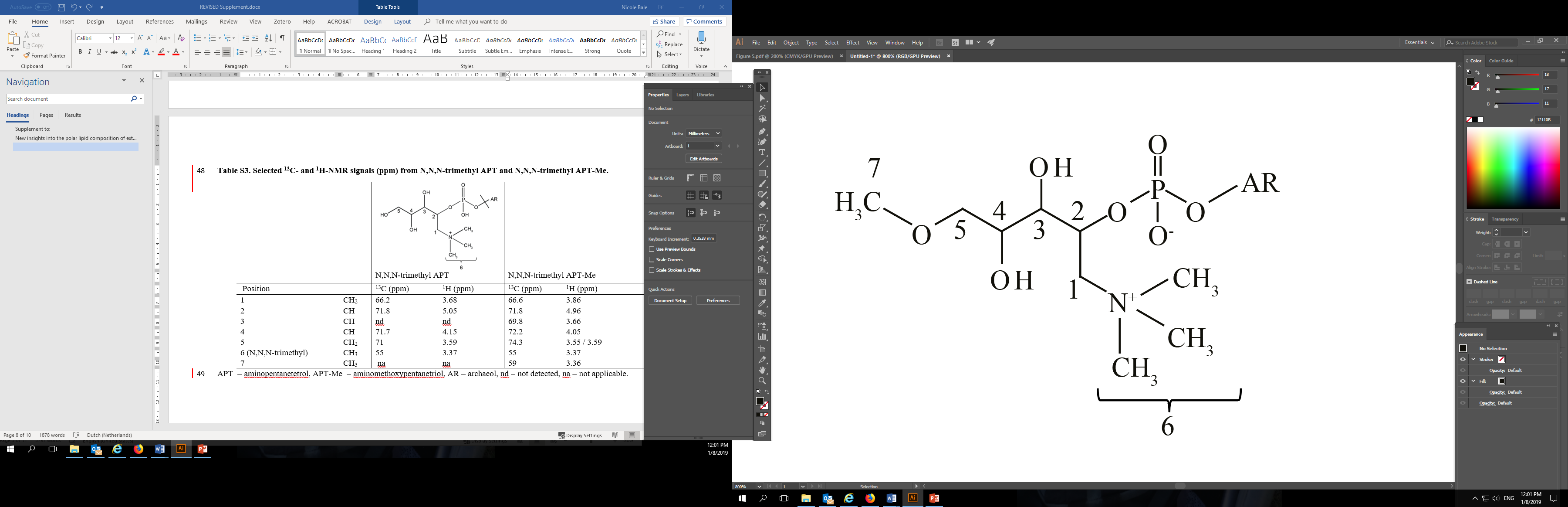 | |
| --- | --- | --- | --- | --- | --- |
|  |  | N,N,N-trimethyl APT | | N,N,N-trimethyl APT-Me | |
| Position |  | ^13^C (ppm) | ^1^H (ppm) | ^13^C (ppm) | ^1^H (ppm) |
| 1 | CH_2_ | 66.2 | 3.68 | 66.6 | 3.86 |
| 2 | CH | 71.8 | 5.05 | 71.8 | 4.96 |
| 3 | CH | nd | nd | 69.8 | 3.66 |
| 4 | CH | 71.7 | 4.15 | 72.2 | 4.05 |
| 5 | CH_2_ | 71 | 3.59 | 74.3 | 3.55 / 3.59 |
| 6 (N,N,N-trimethyl) | CH_3_ | 55 | 3.37 | 55 | 3.37 |
| 7 | CH_3_ | na | na | 59 | 3.36 |

APT = aminopentanetetrol, APT-Me = aminomethoxypentanetriol, AR = archaeol, nd = not detected, na = not applicable.

**Table S4. Accurate masses of the ammoniated molecular ions of the novel lipids IIIa-d and their fragments (UHPLC-HRMS MS^2^).**

|  | **[M+NH_4_]^+^** | **Assigned elemental**  **composition of [M+NH_4_]^+^** | **Δ mmu*** | **MS^2^ product ions** | **Assigned elemental**  **composition of MS^2^ product ions** | **Δ mmu*** | **MS^2^ product ions rationalization** |
| --- | --- | --- | --- | --- | --- | --- | --- |
| IIIa | 1091.7936 | C_55_H_115_N_2_O_16_S | -2.6 | 994.8116  832.7605  653.6800  373.3675 | C_55_H_112_NO_13_  C_49_H_102_NO_8_  C_43_H_89_O_3_  C_23_H_49_O_3_ | -1.2  0.5  -0.6  -0.1 | Loss of SO_3_ and NH_3_  Loss of hexose from 994.812  AR (loss of C_6_H_10_NO_5_ from 832.760)  C_20_ + glycerol |
| IIIb | 1161.8720 | C_60_H_125_N_2_O_16_S | -2.4 | 1064.8916  902.8398  723.7582  443.4470  373.3676 | C_60_H_122_NO_13_  C_54_H_112_NO_8_  C_48_H_99_O_3_  C_28_H_59_O_3_  C_23_H_49_O_3_ | 0.5  1.6  -0.7  1.1  -0.1 | Loss of SO_3_ and NH_3_  Loss of hexose from 1064.891  EXT-AR (loss of C_6_H_10_NO_5_ from 902.839)  C_25_ + glycerol  C_20_ + glycerol |
| IIIc | 1171.7515 | C_55_H_115_N_2_O_19_S_2_ | -1.5 | 994.8140  832.7569  653.6804  373.3675 | C_55_H_112_NO_13_  C_49_H_102_NO_8_  C_43_H_89_O_3_  C_23_H_49_O_3_ | 1.2  -3.1  -0.2  -0.1 | Loss of 2 x SO_3_ and NH_3_  Loss of hexose from 994.814  AR (loss of C_6_H_10_NO_5_ from 832.756)  C_20_ + glycerol |
| IIId | 1241.8306 | C_60_H_125_N_2_O_19_S_2_ | -0.6 | 1064.8915  723.7591  373.3685 | C_60_H_122_NO_13_  C_48_H_99_O_3_  C_23_H_49_O_3_ | 0.4  0.2  0.9 | Loss of 2 x SO_3_ and NH_3_  EXT-AR (loss of hexose and C_6_H_10_NO_5_ from 1064.890)  C_20_ + glycerol |

* (Measured mass – calculated mass) x 1000

**Table S5. Distribution of core lipids in diethers and cardiolipins from HPLC-ITMS analysis. IPLs were examined in terms of their peak area response and thus the proportions reported do not reflect quantitative relative abundance and hence the data is represented qualitatively. See Table 2 for quantitative analysis. See text for abbreviations.**

|  |  | **Methanogens** | | | | **Anaerobic sulfur reducers** | | | | **Polysaccharide utilizers**  **Cellulolytics Chitinolytics** | | | | |
| --- | --- | --- | --- | --- | --- | --- | --- | --- | --- | --- | --- | --- | --- | --- |
|  |  | AMET1^T^ | HMET1 | AME2^T^ | AMF2^T^ | HSR2^T^ | HSR6^T^ | AArc-Mg | AArc-Sl ^T^ | HArcel1^T^ | AArcel5 | AArcel7 | AArcht4^T^ | AArcht-Sl^T^ |
| **Diether core lipids** | **AR** | ++ | ++ | ++ | ++ | ++ | ++ | ++ | ++ | ++ | ++ | + | ++ | ++ |
|  | **Uns-AR** | ++ | ++ | + | ++ | + | Tr | + | + | + | + | + | + | + |
|  | **OH-AR** |  |  | + |  |  |  |  |  |  |  |  |  |  |
|  | **Uns-OH-AR** |  |  | + |  |  |  |  |  |  |  |  |  |  |
|  | **EXT-AR** |  |  |  |  | ++ | ++ | ++ | + | + | ++ | ++ | ++ | ++ |
|  | **Uns-EXT-AR** |  |  |  |  | + | Tr | + | + | + | + | + | + | + |
|  | **MAR** |  |  | Tr |  |  |  |  |  |  |  |  |  |  |
| **Cardiolipin core lipids** | **AR** |  | + |  |  |  |  |  |  |  | + | + | + |  |
|  | **EXT-AR** |  | + |  |  |  |  |  |  |  | Tr | + | + |  |
